# Epidemiological Study of Hazelnut Bacterial Blight in Central Italy by Using Laboratory Analysis and Geostatistics

**DOI:** 10.1371/journal.pone.0056298

**Published:** 2013-02-12

**Authors:** Jay Ram Lamichhane, Alfredo Fabi, Roberto Ridolfi, Leonardo Varvaro

**Affiliations:** 1 Department of Science and Technology for Agriculture, Forestry, Nature and Energy (DAFNE), Tuscia University, Viterbo, Italy; 2 Hazelnut Research Center, Viterbo, Italy; University of Helsinki, Finland

## Abstract

Incidence of *Xanthomonas arboricola* pv. *corylina*, the causal agent of hazelnut bacterial blight, was analyzed spatially in relation to the pedoclimatic factors. Hazelnut grown in twelve municipalities situated in the province of Viterbo, central Italy was studied. A consistent number of bacterial isolates were obtained from the infected tissues of hazelnut collected in three years (2010–2012). The isolates, characterized by phenotypic tests, did not show any difference among them. Spatial patterns of pedoclimatic data, analyzed by geostatistics showed a strong positive correlation of disease incidence with higher values of rainfall, thermal shock and soil nitrogen; a weak positive correlation with soil aluminium content and a strong negative correlation with the values of Mg/K ratio. No correlation of the disease incidence was found with soil pH. Disease incidence ranged from very low (<1%) to very high (almost 75%) across the orchards. Young plants (4-year old) were the most affected by the disease confirming a weak negative correlation of the disease incidence with plant age. Plant cultivars did not show any difference in susceptibility to the pathogen. Possible role of climate change on the epidemiology of the disease is discussed. Improved management practices are recommended for effective control of the disease.

## Introduction

Hazelnut (*Corylus avellana* L.) represents an economically important nut crop of Italy. The country is the second largest producer worldwide after Turkey [1]. In Italy, the production of this crop is concentrated in Campania, Latium, Sicily and Piedmont regions in order of importance [2]. The province of Viterbo has 90% of the hazelnut cultivations of Latium region where Tonda Gentile Romana is the predominant cultivar in over 85% of the orchards [3].

Bacterial blight of hazelnut is caused by *Xanthomonas arboricola* pv. *corylina* (hereafter Xac) [4]. The disease first occurred in the U.S.A. on *Corylus maxima* [5] and further spread in other continents [6], [7], [8], [9], [10]. Recent reports of bacterial blight disease on hazelnut regard the countries like Iran [11], Germany [12], Poland [13] and Chile [14] explaining the movement of the pathogen between the countries *via* propagation materials. However, this disease is not widespread in Europe and as such the European and Mediterranean Plant Protection Organizations included this pathogen in the A2 list of quarantine microorganism [9], [10]. The damage caused by Xac regards mainly young hazelnut plants (1–4 year old) in orchards killing up to 10% [9], [10]. The losses are even more severe in nurseries, where suckering is widely practiced on the mother plants. However, devastating damage can occur also on older (7–8 years) plants [10].

In Italy, Xac was first reported in Latium [15], [16] and successively in Campania regions [17]. During the early nineties, endemic presence of the pathogen was described in central Italy [18]. Recently, hazelnut plants infected by Xac were noticed also from the Italian islands [19]–[21]. Nonetheless, no economically important loss, associated to hazelnut bacterial blight, was reported previously, from central Italy [22].

The current status of the bacterial blight has been changing drastically, for some years now. Frequent occurrence of this disease with severe damage, found across the orchards, was a serious matter of concern by growers. A prime example could be the severe canker symptoms caused by bacterial blight on cv. Tonda di Giffoni [23], the only Italian cultivar that did not bear the canker symptoms in the field for a century [24].

The need to carry out a detailed epidemiological study raised following the outbreaks of this disease across central Italy. Apparently, distribution and incidence of the disease were heterogeneous across the Viterbo province which suggested the possible role of pedoclimatic factors in disease occurrence. Recent finding of the disease in other European countries [12], [13] and the outbreaks in central Italy [23] could in part be associated to the possible effect of climate change. The role of the latter on crop-disease interaction has been a serious matter of concern by many authors [25]–[28]. Regarding the soil, its physical and chemical properties play an important role in the plant health and the consequent disease occurrence and spread [29], [30]. In addition, crop management practices influence significantly the occurrence and control of hazelnut bacterial blight [31].

Spatial patterns of plant pathogens and diseased plants can facilitate the determination of relationships between inoculum density and disease incidence; optimal sampling parameters; the influence of cultural, biological and environmental factors on population dynamics; and the risk assessment of genetically altered microorganisms [32]–[36]. Different methods of spatial pattern analysis are used to characterize the spatial position of plant pathogens and diseased plants [37]–[44]. Spatial autocorrelation functions use the linear correlation between the spatial series and the same series at a further distance interval to detect spatial dependence and have been applied to studies in plant pathology [41], [45], [46].

Geostatistics represent one of the most applied techniques to study spatially related data [47]. Their application has undergone a rapid expansion in plant disease epidemiology and management [48]–[52]. More specifically, spatial pattern analysis has been used to investigate factors affecting plant diseases [53]–[56]. The spread of plant diseases can be estimated by spatial interpolation methods, due to its relation with geographical variables such as soil and climatic characteristics [56]. These techniques were successfully applied to analyze plant disease epidemics even at plot or field scales [33], [57]–[59]. Modelling of the spatial autocorrelation represents a crucial point of geostatistical analysis. This can be performed by examining the variogram estimation. The model variogram is incorporated into a procedure for surface interpolation known as “kriging”. The advantage of the latter is of having two outcomes, a surface map of the variable and a surface map of the kriging standard deviation (KSD). The second provides a relative measure of confidence in the estimates [60]–[63]. Pedoclimatic factors can be considered as regionalized variables and as such they can be investigated by means of geo-statistics and kriging. Furthermore, regression analysis can be applied on frequency distribution of disease incidence in relation to the several spatialized pedoclimatic parameters investigated.

The aims of this study were a) to investigate the current status of the bacterial blight disease and its incidence across the main hazelnut cultivated areas of central Italy and b) to analyze the possible correlation between the disease incidence and the spatial distribution of pedoclimatic factors that can contribute in occurring and spreading of the disease, by means of geostatistics.

## Materials and Methods

### Ethics Statement

No specific permits were required for the described field studies. At each study site, the landowner granted us permission to collect hazelnut samples. The studies carried out during the consecutive years 2010–2012 did not involve endangered or protected species. Field surveys were made across the hazelnut orchards, in the Province of Viterbo, central Italy while laboratory and green house studies were carried out at Tuscia University.

### Description of the study area

**Figure 1:**
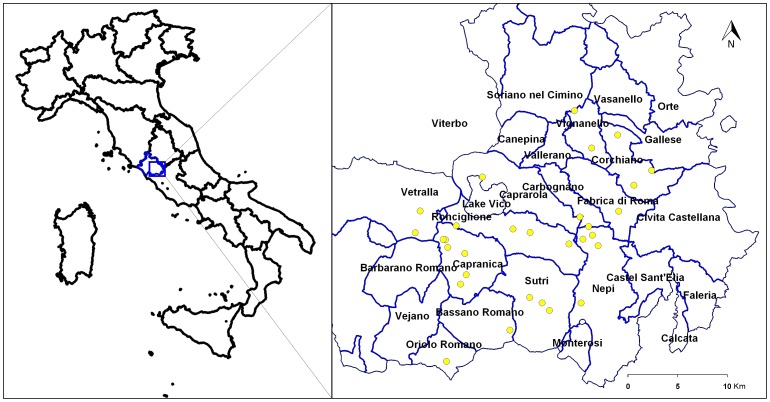
Feature map of the municipalities monitored for bacterial blight. The yellow balls within each municipality represent the number of investigated sites for each municipality, within the province of Viterbo, central Italy.

Viterbo province is located in Latium region, central-western part of Italy between 42.15° and 42.74° north latitude and 11.60° and 12.44° east longitude (Figure 1). Large-scale mechanized production of hazelnut occurs in the province, which is situated in the inner part of the coastal area adjacent to the Tyrrhenian Sea. The topography of the study sites is hilly, and the elevation ranges from 265 to 520 m above sea level (asl). The hill areas where main hazelnut orchards are located are known as “Cimini Hills”.

### Field surveys and data collection

The need to investigate on the current status of the bacterial blight disease, across the hazelnut orchards of central Italy, was based in part on growers report of disease outbreaks in hazelnut fields throughout the province during early summer of 2010 [23].

Twelve municipalities, known for the cultivation of hazelnut, within the province of Viterbo were surveyed (Figure 1). The observations were made over the time period between March and September for all the three years. Each municipality had different number of sites, ranging from a minimum of 1 to a maximum of 6, depending on their extent of hazelnut cultivated area. The size of site varied from 5 to 25 ha and, the total number of sites was 30. From each site, 900 hazelnut trees were randomly surveyed (300/year). Data related to the surveyed municipalities, average age of plant from each site and the cultivars are reported in Table S1. Each site surveyed for bacterial blight infection, was geographically referenced by the Universal Transverse Mercator (UTM) coordinate system [64] with a handheld Global Positioning system (GPS) instrument. The instrument used was a Garmin III Plus (Garmin International Inc., Olathe, KS).

### Sample collection and isolation

**Figure 2:**
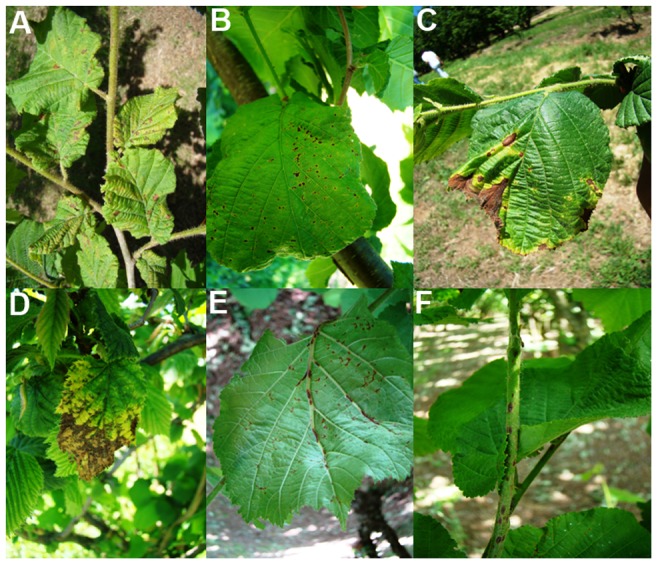
Characteristic leaf symptoms of bacterial blight observed across the hazelnut field in the spring. Water-soaked necrotic spots developed on the leaves at the beginning of the infection (A and B), the presence of extended browning area on the leaves, due to the merging of the necrotic spots, in the late stage of infection (C and D), presence of longitudinal necrosis on the mid-ribs and secondary veins of the lower leaf surface (E) and on young stem (F).

**Figure 3:**
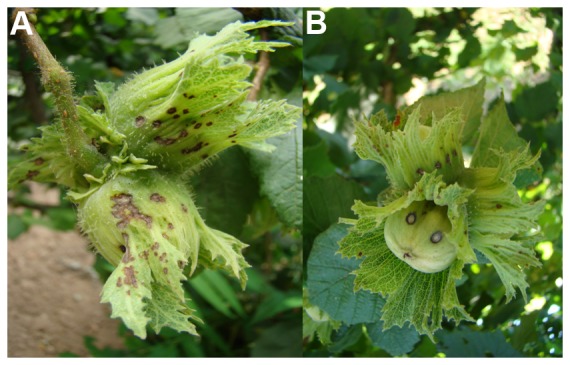
Characteristic symptoms of bacterial blight observed on hazelnut fruit in the field at the beginning of the summer. Oily lesions developed on fruit involucres (A) and fruit shell (B).

**Figure 4:**
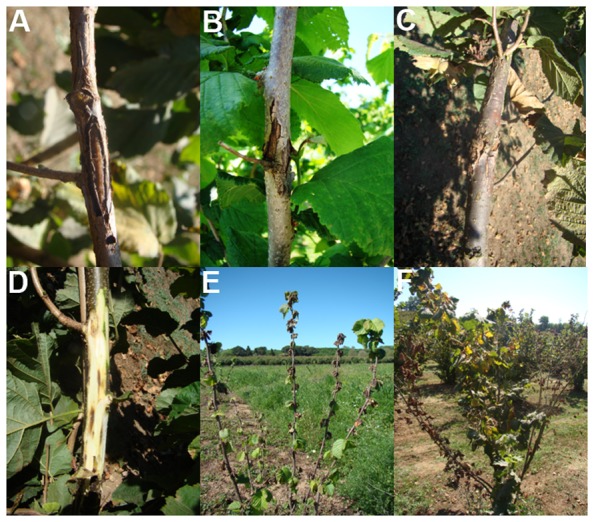
Characteristic symptoms of bacterial blight observed on woody parts across the hazelnut field during late summer. Canker symptoms and cracking of bark tissues, caused by *Xatnthomonas arboricola* pv. *corylina*, on the twigs and branches of hazelnut observed in the field (A, B and C), browning of the sub-cortical tissues (D), wilting of shoots (E) and dieback of branches (F). Figures E and F report the symptoms caused by Xac, rarely found in the field and very similar to those caused by *Pseudomonas syringae* pv. *avellanae*.

Samples were collected over the time period between March and September for all the three years. Plant parts showing characteristic symptoms of bacterial blight (Figures 2, 3 and 4), as those reported in literature [6], [9], [17], [18], [22], [23], [31], [65], [66], were cut, separately put into sterile plastic lab bags and brought to the laboratory. The pruning shears, knife and hand-saw used to cut the samples were sterilized, each time the sample was taken, by immerging them in copper solution for 2 min followed by two rinses in sterile distilled water (SDW), each for 1 min. The number of the samples taken from each site ranged from 15 to 60, depending on the presence and incidence of bacterial blight. In fields where the disease symptoms were sporadic, only few samples were collected within an approximate radius of 100 m. In fields where the disease incidence was high, samples were collected from several neighboring plants. This sampling scheme was adopted to obtain more bacterial isolates possible from different diseased tissues of a given site in order to further investigate the possible phenotypic differences among them.

Immediate sample processing was made, for the isolation of the causal agent. The samples were surface sterilized in 1% NaOCl for 1 min followed by two rinses in SDW (each for 1 min), excised and each section was then crushed in SDW, left for 5 minutes, in order to allow bacterial streaming [23]. Streaking was made by taking a loopful of the resulting suspension onto the surface of the following media: nutrient agar supplemented with 5% sucrose (NAS), yeast extract-dextrose-calcium carbonate agar (YDCA), glucose yeast extract calcium carbonate agar (GYCA) and yeast extract bacto peptone glucose agar (YPGA) media [10], [14], [23]. The plates were incubated at 26±1°C and examined daily up to three days for bacterial growth.

### Disease incidence

One thousand hazelnut trees/site were randomly analyzed each year (total of 3000 trees/site) for the presence of bacterial blight on leaves, stems, fruits, twigs and branches. Any symptoms suggestive to bacterial blight, based on the literature review, were taken into consideration. The incidence of the disease was calculated by the proportion of diseased plants within the total [67]. Statistical analysis were performed using software package Statistica ® (StatSoft, Inc., Tulsa, USA). Student's t test, Pearson correlation and regression analysis were performed to determine the effect of plant age and pedo-climatic factors on disease incidence (DI). However, the influence of plant cultivars on DI was not evaluated for the fact that over 85% of the cultivation in Viterbo is represented by cultivar Tonda Gentile Romana and the remaining are grown in different proportions from one site to another. Moreover, the aforementioned cultivars are the clonal populations derived from the asexually multiplied local hazelnut accessions and as such may not present genetic differences.

### Characterization of the isolates

A consistent number of bacterial isolates were obtained from the diseased tissues sampled across the sites. The colony morphology was evaluated on the media described above. The shape, size, color, margin and pigment were considered. In addition, the ability of the isolates to metabolize succinate-quinate was tested on SQ medium [68]. Ten isolates from each site were used for the biochemical and nutritional tests prior to their identification. One recommended strain, NCPPB 2896 [10] and strains (Xaco1, Xaco2) isolated recently from central Italy, were used as reference [69]. Successively, the isolates were tested for the production of levan, oxidase, pectinolytic enzyme, urease, indole, catalase, lecithinase, tobacco hypersensitivity, metabolism test, H_2_S from peptone and cysteine; hydrolysis of gelatin, aesculin, arbutin and starch; lypolisis of tween 80; tyrosinase activity; tolerance to 0.1% 2,3,5-triphenyltetrazolium chloride (TTC); maximum growth temperature; growth in 2% and 5% NaCl and litmus milk reaction [70], [71]. In addition, growth ability by using different carbohydrates as sole carbon source was tested [72].

### Lesion tests

The ability of the isolates to induce the hypersensitive defense reaction (HR) was tested on bean pods [73]. The hypersensitive reaction to infection of bean pods by the plant pathogenic Xanthomonads, except those pathogenic to bean, was considered as an indicator of bacterial plant pathogenesis [74]. *Pantoea agglomerans* (strain Pag1) was used as negative control [69].

### Pathogenicity tests

Two-year-old, healthy potted hazelnut plants (cvs. Tonda Gentile Romana and Tonda di Giffoni) were inoculated with 30 isolates (one from each site) obtained during our study. The inoculum containing 10^6^ cfu·mL^−1^ was prepared as described previously [23]. Two different inoculation techniques were used on different plants. In the first case, leaves were spray inoculated (10 mL per plant) with a sprayer containing the bacterial suspension. All the plants were covered with plastic bags from 2 hrs before until 2 hrs after spraying to maintain a high relative humidity (90 to 100%). In the second case, wounds made on the woody parts of the plant (twig and branch) were drop inoculated (0.10 µL per wound). The wounds were then sealed with parafilm (Pechiney Plastic Packaging, Chicago, IL, USA) for three days. For each isolate, eight plants were used (4 plants for each cultivar, 2 plants each inoculation technique). Aforementioned reference strains and the SDW were used respectively as positive and negative controls on the same number of plants. The plants were maintained in the greenhouse. The inoculated plants were inspected weekly until the symptoms appeared.

### 16S rDNA gene sequencing

Bacterial cultures for DNA extraction were grown on NA medium for 24 h at 26±1°C. DNA was extracted with PureLink^tm^kit (Invitrogen, Carlsbad, CA, USA) following manufacture's instruction. The purity and integrity of DNA was determined by 1% agarose gel electrophoresis. The concentration of DNA was measured by Qubit^tm^ fluorometer (Invitrogen, Carlsbad, CA, USA), and the final concentration was adjusted to 60 ng/µL.

Molecular identification was achieved by sequencing the complete 16S rRNA gene. To this purpose we used the universal primers NOC1F (AGA GTT TGA TCA TGG CTC AG), NOC1R (GTA TTA CCG CGG CTG CTG GCA C), NOC3F (GCA TGG CTG TCG TCA GCT CGT G) and NOC3R (ACG GTT ACC TTG TTA CGA CTT). The PCR reaction was prepared by mixing 1 µL of 60 ng concentrated template DNA, 1 µL of each primer at 10 µM and 12 µL of DNA Polymerase master mix (GoTaq®Flexi, Promega). The final volume was adjusted at 25 µL by adding SDW. The PCR reactions were performed according to the following program: 1 cycle at 95°C for 5 min., 35 cycles consisting of 30 sec. at 95°C, 1 min. at 55°C and 1 min. at 72°C and 1 cycle (final elongation) at 72°C for 4 min.

The PCR product was loaded on 1% agarose gel electrophoresis to determine the presence and size of the bands. The bands at the expected size were sequenced, analyzed with chromas version 1.45 (32-bit) and aligned with other sequences present in the Genebank by using CLUSTAL W software available online. The similarity of our sequences was compared with those of reference strains present on NCBI by blasting and the sequences were deposited in GeneBank.

### Climatic and environmental data

**Figure 5:**
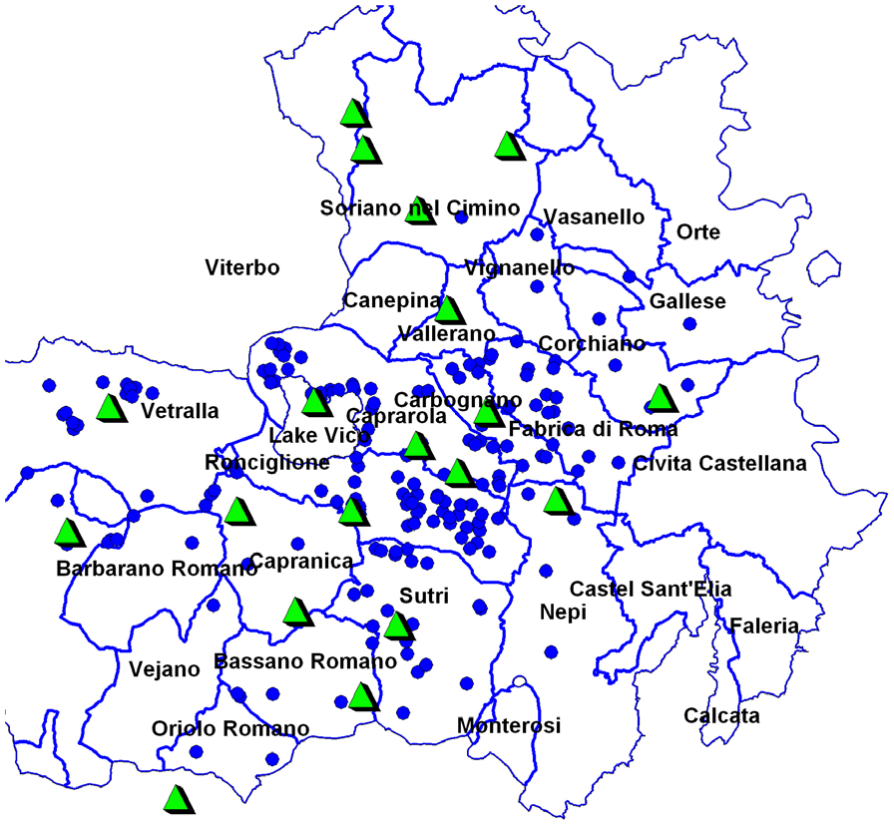
Feature map showing the locations from where pedoclimatic data were collected. Soil analyzed areas (blue beads) and meteorological stations (green triangles) across the hazelnut sites of Viterbo province.

The meteorological data recorded hourly by different stations, located around the Cimini Hills, were collected. Temperature data (°C), only of the last ten years, were recorded by 19 stations across the province. Regarding the rainfall (mm), data collected from 1970 to 2012 from several meteorological stations, most of them not active any longer, were considered. In addition, a database of soil chemical-physical analysis of more than 180 geo-referred sites in the area was set up (Figure 5). Besides meteorological data, some essential soil parameters contributing to the presence and spread of the disease were investigated. In particular, the effects of rainfall [75], total Nitrogen amount (%) in the soil [76], [77], soil Mg/K ratio were evaluated. The latter is of considerable importance that explains certain specific Mg deficiency *in planta*, especially on acidic soils [76]. Our study sites, characterized by the volcanic soils, have this deficiency which might increase plant susceptibility to various diseases [78]–[80]. The influence of lower pH associated to soil aluminium (meq/hg) was investigated given its influence in plant-pathogen interaction [81]–[83]. Finally, the average thermal shock (Δ), given by the difference among the day/night temperature, associated to every frost event recorded was calculated. The latter was found strictly associated to dieback of hazelnut, caused by *Pseudomonas syringae* pv. *avellanae*, in the same area [84]. All the values were spatialized according the geostatistical methods as described above. Kriging was applied to the real or log-transformed data. An exception was made for the average rainfall data given that they were collected in a period of about forty years since 1970 from several meteorological stations, most of them not active any longer. In particular, rainfall map consisted in a vectorial filled contour layer made up of interpolated level curves with an interval of 1 mm of rainfall. The level curves traced were at a distance of 100 mm for simplicity although the actual resolution was of 1 mm. Estimated meteorological and pedo-chemical parameters, together with their associated standard error (SE), were then correlated with the average DI for each site by means of Regression Analysis (RA). In all the correlation graphs the real DI is expressed in logarithmic scale, thus the correlations between the incidence itself and the various parameters are straight-line logarithmic correlations.

The analyses were performed on the incidence averaged over the three years rather than on each temporal observation independently. Previous preliminary tests conducted on the seasonal epidemiological data, of bacterial canker and bacterial blight of hazelnut, in relation to the seasonal rainfall trend in Viterbo showed no significant correlation at year level. However, a significant correlation was found between the average rainfall of at least 10 years and the incidence of bacterial canker caused by *P. avellanae* [84]. Additionally, the spread of the bacterial diseases seemed very less correlated to specific rainfall events which may occur throughout the territory as a rainstorm over the years. Indeed, the disease does not spread with a particular way across the territory, within the single year characterized by particular meteorological events or intense periods of drought, remaining confined to areas more or less circumscribed [85]. All these data suggest that incidence averaged over the three years is much more reliable compared to the single temporal observation, especially when the range of the sites is wide.

### Geostatistical analysis and kriging of the meteorological and pedological data

In order to obtain a basic knowledge of the data set, conventional statistical analysis of the pedoclimatic data was prior conducted using software package Statistica ® (StatSoft, Inc., Tulsa, USA). Student's t test, Pearson correlation and regression analysis were performed to determine the correlation of pedoclimatic factors with DI. Prior to conduct geostatistical analyses, on the spatial patterns of data collected from the meteorological stations and sites of Cimini Hills, the latitude and longitude coordinates of the points were first transformed into plane coordinates with a Universal Transverse Mercator (UTM) projection, Zone 33N, European Datum 50, in ArcGIS (version 8.0, ESRI, Redlands, CA). The UTM coordinates were used in the subsequent statistical analyses. The UTM system gives the positions in meters and is preferred over latitude-longitude in decimal degrees. The position coordinates in meters facilitate the correct computation of distances between sample locations in a plane (two dimensions), essential in a geostatistical analysis. Geostatistical results are presented as semivariograms, which represent the average of squared differences in values between pairs of samples separated by a given distance [86]. Semivariograms are statistical measures that assume normally distributed input sample data where local neighbourhood means and standard deviations show no trends [42], [43]. Statistically, abnormal distribution of pedoclimatic data can have an adverse impact on semivariogram analysis and further interpolation of data sets. To moderate this effect, the logarithmic transformation was also applied to measured pedoclimatic data before geostatistical analysis [60], [61]. Coefficient of skewness (Cs) and coefficient of kurtosis (Ck) are calculated for pedoclimatic data, before and after the logarithmic transformation. Although transformed data cannot pass relative test for normal distribution, they obviously obtained lower Cs and Ck in comparison with those untransformed ones. Semivariogram calculation was then conducted with log-transformed data.

The experimental variograms of the data were fitted by a spherical model with different ranges and sill variances. Eight lags, with a lag distance of 1600 m, were used for the semivariogram calculation, then the model fitting give us key parameters describing spatial structure of data. These parameters include the Nugget value (N), Partial Sill (pS), Sill (S = N+pS), and Range value. Sill is an estimated semivariance that marks where a plateau begins. The Nugget value presents the variability at zero distance (spatial random variance of regional variable). The pS value presents the sill proportion of explainable semivariance. The Range is defined as the separation distance corresponding at about 95% of the Sill [60], [61]. The N/S ratio (NSR) was selected to express short-distance autocorrelation of regionalized variables [87]. Low NSR indicates high spatial autocorrelation or spatial continuity over short distances. The spatial dependence was defined using the nugget to sill ratio convention [88], whereby nugget/sill <0.25, >0.25–<0.75 and >0.75 corresponds to strong, moderate and weak spatial dependence, respectively.

The theory of regionalized variables [89] was applied. The objective was to model and identify the spatial structure of the variable and to estimate its values across the studied area. Linear and nonlinear models were fitted to semivariograms by least squares regression using Geostatistical Analyst module of ArcGIS (ESRI). The pedoclimatic variables modelled were regressed on distance with or without logarithmic transformation. Successively, the Gaussian, spherical, linear, linear to sill, and exponential models, were used to describe semivariograms [90]. The coefficient of determination (r^2^), structural variance (proportion of spatial structure), and mean square error (MSE) were used to evaluate the goodness-of-fit of data and to choose the best regression model [91]. Anisotropy was determined by comparison of semivariogram characteristics. Oriented semivariograms that displayed differences among semi-variogram characteristics for different directional orientations indicated anisotropy or directionality in the degree of spatial dependence [86]. Ordinary kriging was applied to estimate logarithmic values of pedoclimatic data for each of 19 meteorological stations or 180 soil analysis sites. In addition, vectorial maps were gained using anti-logging calculation. The whole application, including semi-variogram calculation and kriging (ArcGIS version 8.0, ESRI, Redlands, CA, USA), was applied to log-transformed data. The reliability of the applied model was tested by cross validation between measured and interpolated values. Interpolated data have been depicted as colored raster maps, limiting the prevision to the areas where data were collected.

## Results

### Disease symptoms in the fields

Initial symptoms began to appear on leaves, during the early spring, when the phase of leaf development was terminated and the temperature was more favorable to leaf infection. Also the stem and new shoots were affected over time. The woody parts of the plants were asymptomatic until the late spring but the symptoms began to appear afterwards.

Water-soaked necrotic spots (Figure 2 A and B) appeared on the leaf surfaces. Initially, only few spots were seen but their number increased from March to September and over the sampling years 2010–2012. The type, the number and the size of the spots varied significantly within and among the orchards (Figure 2). Often, leaves showed numerous oily polygonal lesions which merged together causing a general chlorosis of the lamina. Infected leaves sometimes showed browning of the leaf margins (Figure 2 C and D). Longitudinal browning and necrosis along the mid-ribs and secondary veins on the lower leaf surface were frequently found (Figure 2 E). In some cases, even the lesions on the new stems can be seen (Figure 2 F). The dieback of new lateral shoots was common. During the summer, black heel symptoms with the browning of the shell and corresponding part of the involucres were observed on the fruits. In addition, oily lesions of different size (2–6 mm long) can be seen, on the involucres and shell before lignifications (Figure 3 A and B). The formations of 10–20 cm long cankers, with longitudinal cracking of the bark, were seen along the branches and main trunks, especially on young plants (Figure 4 A and B). Moreover, the appearance of brownish and/or black necrosis was observed over time on the convex side of the layered branches and shoots (Figure 4 C). When the bark of the infected branch was excised, the necrosis was visible also on the cortical tissues (Figure 4 D). Interestingly, affected twigs were often bent and the swelling was very common on the bent parts. In the late summer, shoot wiltings and necrosis (Figure 4 E) was spread to the trees stump causing the complete dieback of one or more branches (Figure 4 F).

Leaf spots and new shoots dieback were equally present both on the plants younger than 6-year old and those having older age. The presence of canker and the dieback of one or more branches can be seen only on the plants younger than 6 years. All the examined cultivars were infected.

### Disease incidence

**Figure 6:**
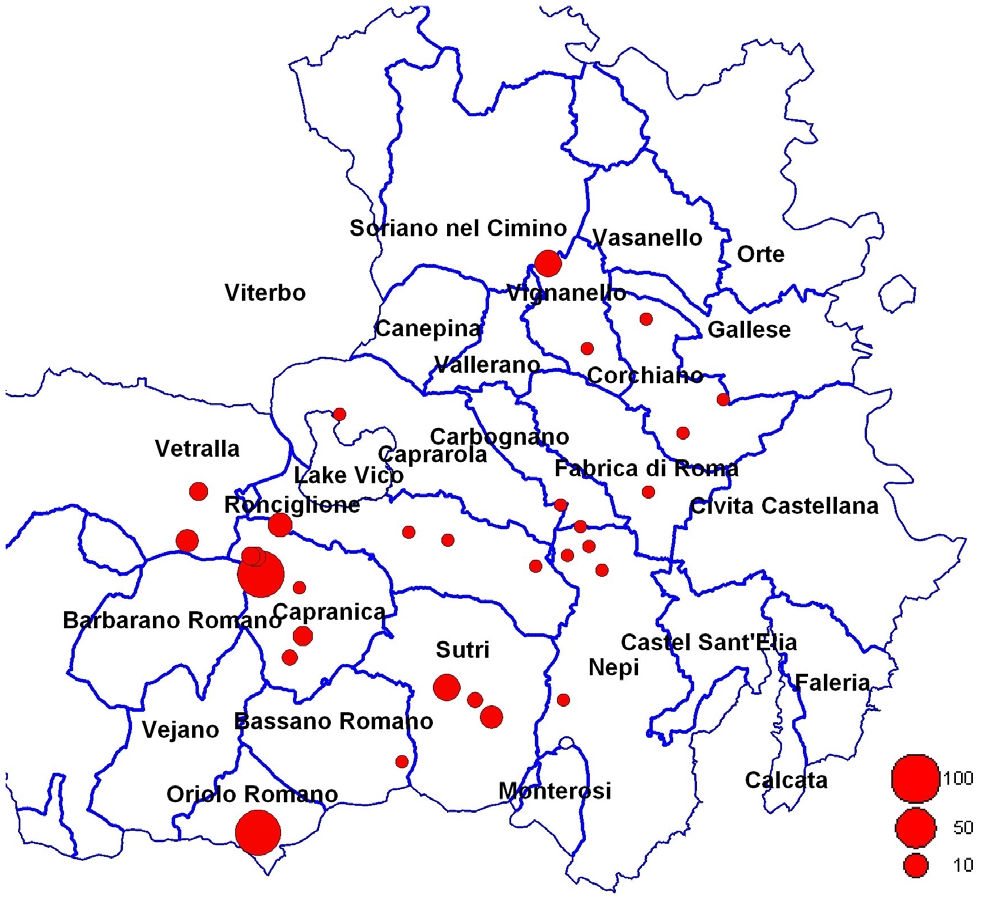
Incidence of the bacterial blight (%) across the study sites in the province of Viterbo. The red circle size inside the map indicates the different disease incidence expressed in logarithmic scale.

The incidence of the disease ranged from <1% to almost 75% across the study sites (Figure 6, Table S1). All the surveyed sites presented the characteristic symptoms on the herbaceous parts of the plant whereas the canker symptoms and the consequent dieback of one or more branches were present only on young plants. Equal to the plant age, disease incidence varied from site to site (Table S1). Disease incidence and plant age ahowed a weak negative correlatin (t = −3.74; P<0.001; r^2^ = 0.33)(Appendix S1).

### Identification of the isolates

Yellow-mucoid, shiny and rounded bacterial colonies were observed on NAS, YDCA, GYCA and YPGA media after 72 hrs of incubation. The colonies were of approximately 2.5 to 3 mm in diameter on NAS but they were slightly smaller on the other media. All of them showed oxidative metabolism and produced the HR on tobacco leaves and bean pods. The isolates were negative for gram, oxidase, indole, lecithinase, urease, tyrosinase, nitrate reduction, growth in 5% NaCl, lypolisis of tween 80 and positive for catalase, growth in 2% NaCl, hydrolysis of gelatin, esculin, arbutin and starch, growth at 35°C and production of H_2_S from peptone and cysteine. In addition, all of them gave alkaline reaction to litmus milk, tolerated to 0.1% TTC and produced dark green and yellow pigments on SQ and YDCA media respectively. The isolates used glycerol, D-mannitol, fructose, galactose, trealose, sucrose, glucose, maltose, L-asparagine, D-sorbitol, L-arginine, salicin and inulin as sole carbon sources and caused their acidification except of the last four. All of them were negative for the utilization of L(+) tartrate.

On the basis of the results obtained from the morphological, physiological, biochemical and nutritional tests, the isolates causing the aforementioned symptoms were identified as *Xanthomonas arboricola* pv. *corylina*. The comparison of our sequences with those present in the database further confirmed the results regarding the belonging of our isolates. Our sequences (Accession numbers JQ861273, JQ861274 and JQ861275) shared 99% identity with the analogous sequences of Xac available in the NCBI database.

### Lesion tests

All Xac isolates produced water-soaked lesions at the site of inoculation of bean pods, after 4 days. No symptoms were observed on those inoculated with *P. agglomerans* (Figure S1).

### Pathogenicity tests

All the isolates reproduced the symptoms of the disease on the plants artificially inoculated. Water-soaked necrotic spots (Figure S2 A) followed by shoot witlings (Figure S2 B) were observed on leaves within a month after the artificial inoculation. By contrast, canker symptoms began to appear only after a month (approx. 5 weeks) (Figure S2 C). The canker observed on the artificially inoculated plants were smaller (2–4 cm) than those observed in the field. Results showed that both of the cultivars are similarly susceptible (t = 0.59; P = 0.64; r^2^ = 0.23) to the pathogen. No significant difference (t = 1.38; P = 0.43; r^2^ = 0.17) in time of symptom appearance was found among the isolates and reference strains. Plants inoculated with the reference strains produced the same symptoms whereas control plants inoculated with SDW remained healthy. Bacteria re-isolated from the diseased parts of the plants had the same characteristics of the inoculated strains.

### Statistical analysis and kriging of the meteorological and pedoclimatic data

Details on the type of distribution (leptokurtic and platykurtic) of pedoclimatic data and the difference between normal and log-transformed data are provided in Appendix S2.

Table 1 shows the semivariogram parameters of the spherical model applied to the pedoclimatic data. The spatial dependence ranged from moderate (for the total nitrogen and Mg/K ratio) to high (for the thermal shock). In particular, total nitrogen data had an N/S ratio of 0.636, inferring moderate spatial dependence. This means that 36.4% of the total variation in total nitrogen present can be explained by spatial variations while the remaining 63.6% was attributable to unexplained sources of variations. For thermal shock, an N/S ratio of 0.153 was indicative that 84.7% of total variation was spatial variation while only 15.3% was due to other sources of variation. The spatial dependence was moderate also for soil aluminium and pH (Table 1).

**Table 1:** Semivariogram parameters object of study.

Parameter	N	pS	N/S ratio	Range (m)
**Total Nitrogen**	0.116	0.066	0.636	12649
**Mg/K ratio**	0.082	0.102	0.446	5635
**Δ**	0.005	0.026	0.153	10335
**Aluminium**	1.222	1.592	0.434	12648
**Soil pH**	0.008	0.005	0.611	12650

N: nugget; pS: partial Sill; S: sill; N/S ratio = [N/(N+pS)]; Δ: thermal shock.

Summary statistics of cross validation prediction errors (CVPE) applied to Log data are shown in table 2. Here the term “prediction error” was used for the difference between the prediction and the actual measured value. Cross-validation provides indexes useful to determine the goodness of the model used in the study. For a model that provides accurate predictions, the mean prediction error (MPE) should be close to 0 if the predictions are unbiased, the standardized root-mean-square prediction error should be close to 1 if the standard errors are accurate, and the root-mean-square prediction error should be small if the predictions are close to the measured values. The goal should be to obtain the standardized mean prediction errors (SMPE) close to 0, the small root-mean-square prediction errors (RMSPE) and the average standard error (ASE) near root mean square prediction error (RMSPE); and the standardized root-mean-square prediction errors (SRMSPE) close to 1.Among the investigated parameters, the best fitting model was found for total nitrogen followed by soil pH, Mg/K ratio, thermal shock and then for aluminium (Table 2).

**Table 2:** Summary statistics of cross validation prediction errors applied to log-transformed data.

Parameter	Mean	RMSPE	ASE	SMPE	SRMS
**Total Nitrogen**	0.000089	0.052660	0.053480	−0.002782	0.975300
**Mg/K ratio**	−0.012700	0.349200	0.292900	−0.059400	1.235000
**Δ**	−0.134000	1.553000	1.883000	−0.051540	0.825100
**Aluminium**	0.106900	0.908300	4.045000	0.020600	0.283700
**Soil pH**	−0.007366	0.561900	0.537800	−0.011860	1.040000

RMSPE: root mean square prediction error; ASE: average standard error; SMPE: standardized mean prediction error; SRMS: standardized root mean square; Δ: thermal shock.

Appendix S1 reports the correlation results of statistical tests, p-values and Pearson correlation coefficient matrixes of the logarithmic disease incidence (Log DI) with pedoclimatic variables and plant age. The degree of freedom for the correlation analysis (Appendix S1; Table 1), Pearson correlation analysis (Appendix S1; Table 2) and regression analysis (Appendix S1; Table 1) are 28, 22 and 7.22, respectively. Values showed a strong positive correlation of DI with average rainfall (t = 11.54; P<0.001; r^2^ = 0.82), thermal shock (t = 11.65; P<0.001; r^2^ = 0.82) and average soil nitrogen (t = 17.34; P<0.001; r^2^ = 0.91). Additionally, the correlation was strong negative for Mg/K ratio (t = −10.64; P<0.001; r^2^ = 0.80) and weak positive for aluminium (t = 3.99; P<0.001; r^2^ = 0.36). No correlation of DI was found with soil pH (t = −1.10; P = 0.28; r^2^ = 0.04).

**Figure 7:**
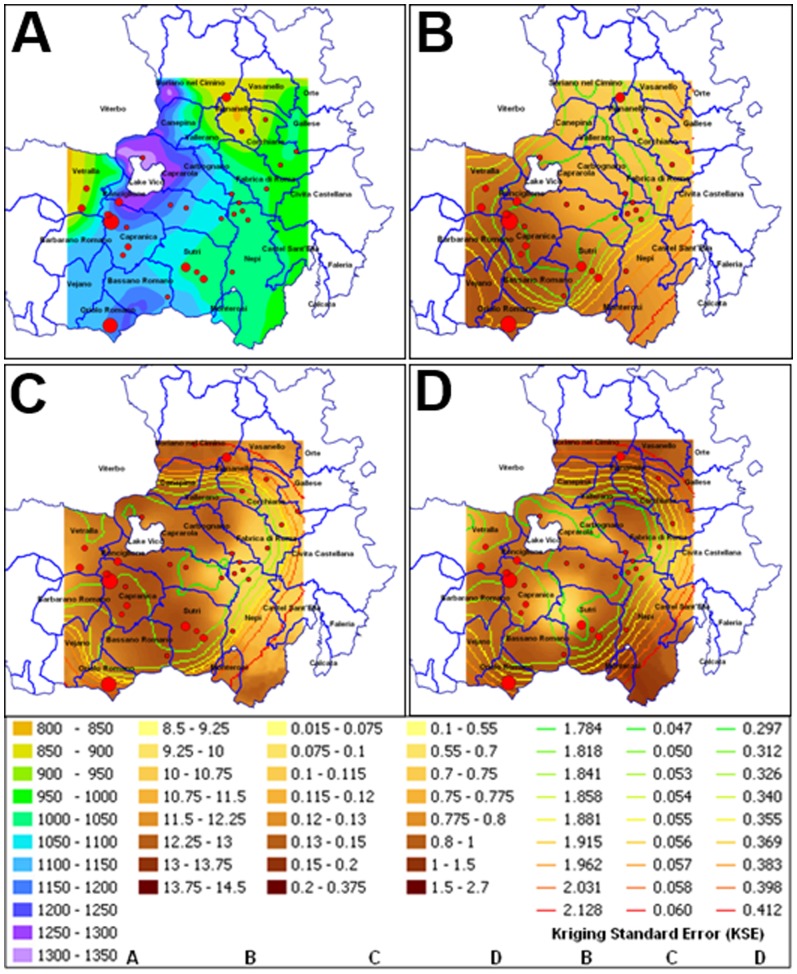
Map illustrating the correlation of average disease incidence (%) with different pedoclimatic factors. Average rainfall values (A), kriging of thermal shock associated with frost (B), kriging of nitrogen content in the soil (C) and kriging of Mg/K ratio in the soil (D). The values are expressed in the following units: rainfall (mm), thermal shock (Δ = °C) obtained from the difference between maximum and minimum daily temperature, content of nitrogen in the soil (%) and the last is a ratio between magnesium (Mg) and potassium (K) content in the soil. The red circle size inside the maps indicates the different disease incidence (<1% to 75%) expressed in logarithmic scale.

**Figure 8:**
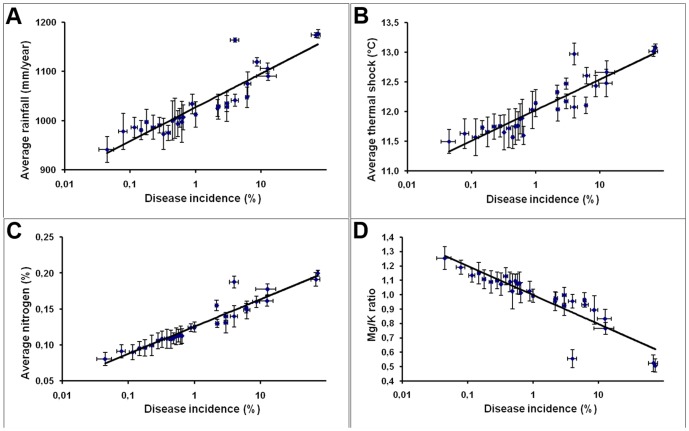
Correlation of the average disease incidence (%) of each site with different pedoclimatic factors. (A) average rainfall values (t = 11.54; P<0.001; r^2^ = 0.82), (B) thermal shock associated with frost (t = 11.65; P<0.001; r^2^ = 0.82), (C) nitrogen content in the soil (t = 17.34; P<0.001; r^2^ = 0.91) and (D) Mg/K ratio in the soil (t = −10.64; P<0.001; r^2^ = 0.80). The horizontal and vertical bars represent the standard error of disease incidence and pedoclimatic variables, respectively. Six were the average number of replicate in each site (n = 6).

The incidence of bacterial blight, expressed in logarithmic scale, in relation to the average rainfall data of the last 40 years, are reported in Figure 7A. The incidence appeared well correlated with the higher average annual rainfall (1000–1300 mm rainfall classes of rain per year). The correlation was further confirmed by linear regression analysis (r^2^ = 0.82) (Figure 8A, Appendix S1).

The thermal shock values (in °C) in correspondence to the disease incidence are shown in figure 7B. The incidence increased across the areas where these values ranged between 13 and 15°C. The linear regression between the data of the heat shock, estimated with the logarithm of the real values of the disease incidence confirmed the correlation. The significance of the correlation itself was confirmed by the value of r^2^ equal to 0.82 (Figure 8B; Appendix S1). The change in temperature was divided into eight classes covering a distance of values between 8.5 and 14.5°C, which are also associated with a kriging standard error (from 1.784 to 2.128).

The estimated total amount of nitrogen, divided into eight classes, and the incidence of bacterial blight are demonstrated in Figure 7C. The lowest class corresponded to an estimated content of nitrogen that varied from 0.015 to 0.075% up to the highest class, where the content of this element was between 0.21 and 0.375%. A kriging standard error (ranging from 0.047 to 0.06) was associated to the prediction of the amount of total nitrogen in the soil. A direct linear relationship between the nitrogen contents and the logarithm of the disease incidence was confirmed by the linear regression value (r^2^ = 0.91) (Figure 8C; Appendix S1). A low global standard error was observed for this correlation which is able to better discriminate the DI per site in relation to the nitrogen content. The Mg/K ratio in the soils, divided into eight classes, are reported in Figure 7D. The ratio ranged from the minimum of 0.1 up to the maximum of 2.7. For each of these values a standard error (between 0.297 to 0.412) was associated. At low Mg/K ratios the disease incidence was greater showing an inverse correlation. The correlation was confirmed by analysis of linear regression with an r^2^ value of 0.80 (Figure 8D; Appendix S1).

Pearson correlation values among each pair of variables are reported in Appendix S1. Results showed that nitrogen was negatively correlated with Mg/K ratio (P<0.001; r = −0.95) and plant age (P<0.001; r = −0.54) while it was positively correlated with thermal shock (P<0.001; r = 0.95), rainfall (P<0.001; r = 0.95) and aluminium (P<0.001; r = 0.68). Likewise, Mg/K ratio was negatively correlated with thermal shock (P<0.001; r = −0.92), rainfall (P<0.001; r = −0.96), soil aluminium (P<0.001; r = −0.68) and positively correlated with plant age (P<0.001; r = 0.62). Equally, a negative correlation of thermal shockwith plant age (P<0.001; r = −0.48) and a positive one with rainfall (P<0.001; r = 0.93) and soil aluminium (P<0.001; r = 0.64) were observed. Regarding the rainfall, its correlation was negative with plant age (P<0.001; r = −0.59) and positive with soil aluminium (P<0.001; r = 0.65). Finally, a moderate negative correlation of soil aluminium (P<0.01; r = −0.49) was observed with plant age whereas there was a weak positive correlation (P<0.05; r = 0.38) among soil pH and plant age. It is important to note that soil pH did not show any correlation with the pedoclimatic factors analyzed except the weak positive correlation found with plant age as described above.

Results of multiple regression analysis are shown in Appendix 1. Only the regression coefficients for nitrogen were highly significant (β = 1.20; t = 4.83; P<0.001). It means that the null hypothesis (H_0_: β_i_ = 0 for all i, where β_i_ = regression coefficient for every factor) can be rejected for this value. By contrast, the regression coefficient for Mg/K ratio (β = 0.36; t = 1.55; P = 0.13), thermal shock (β = 0.03; t = 0.16; P = 0.87), rainfall (β = 0.06; t = 0.29; P = 0.77), aluminium (β = −0.10; t = −1.34; P = 0.19), soil pH (β = −0.03; t = −0.49; P = 0.62) and plant age (β = −0.13; t = −1.75; P = 0.09) were not significant and as such the null hypothesis cannot be rejected.

The correlation maps of DI with soil aluminium content and pH, reported into eight classes, are shown in Appendix S3. The values of linear regression analysis demonstrated a weak positive correlation between the DI and soil aluminium content (r^2^ = 0.36) whereas no correlation of DI was found with soil pH (Appendix S1; Appendix S3). . Moreover, in both cases, the associated standard error of the two pedological parameters was found to be generally higher for all the sites if compared to the other cases described above (Appendix S3).

## Discussion

Our study reports some important results which are significant to explain the role of pedoclimatic factors in the occurrence and spread of bacterial blight disease. We did not observe phenotypic and pathogenic differences among Xac isolates, obtained during the years 2010–2012. This indicates that different DI across our study sites might not be related to the different degrees of virulence of the isolates. In addition, equal susceptibility of hazelnut cultivars to artificial inoculations further confirms this hypothesis. However, It should be emphasized that hazelnut grown in the Latium region (cvs. Tonda Gentile Romana, Tonda di Giffoni and Nocchione) are the clones derived from local cultivars which are even similar to Spanish ones [92]. The aforementioned cultivars are the result of asexual multiplication carried out by farmers, in practicing traditional agriculture system, in the years sixties-eighties [3], [93]. The lack of genetic breeding programs of this crop resulted in the diffusion of the same clones over the years across Italy by increasing crop vulnerability to biotic and abiotic stresses [94]. The true variability in infectivity profiles can be detected only by using different host cultivars, not existing yet in Italy. A previous detailed study on the genetic and phenotypic characterization demonstrated that Xac populations of different geographic origin, including that of central Italy, are homogeneous [95] which is in full agreement with the results of our phenotypic tests.

Recent disease outbreaks on cv. Tonda di Giffoni in the province of Viterbo with the presence of longitudinal canker might have an explanation. “Tonda di Giffoni” is a typical cultivar grown in Campania region, which is located to the southern part of Latium region having a mild winter temperature and rare spring frosts [24]. Indeed, the presence of bacterial blight symptoms on leaves and sprouts was described from Campania region but canker formation did not occur [24]. The cultivar has been introduced very recently by growers in Latium, a region with cold winter temperatures and frequent spring frosts [84], [96], because this cultivar is characterized by a vigorous growth and an early production. Introduction of this cultivar to areas of Latium region may have predisposed trees to infection by Xac which has an epiphytic phase and enters and cause the canker through wounds [7]–[10].

Concerning the relationship of plant age with the DI caused by Xac, no strong correlation was found among these parameters. However, the DI was weak and negatively correlated to the plant age confirming that young plant are the most affected by the disease as previously reported [9], [10]. Geostatistical analysis showed different levels of spatial autocorrelation for spatialized parameters. The best autocorrelation was found for the thermal shock (N/S ratio<0.25). The other two parameters showed less spatial autocorrelation (N/S ratio between 0.25 and 0.75). In all cases, the spherical model adopted results to producing good CVPE [97]–[99], especially for total nitrogen.

In recent years, a large number of studies were made on plant disease epidemics using spatial pattern analysis in order to relate the observed characteristics of epidemics to the underlying ecological and pathological processes [100]–[102]. However, specific studies on the spatial pattern analysis of the pedoclimatic factors affecting plant diseases are lacking in the literature. Often, the role of pedoclimatic factors on the occurrence and spread of the diseases is ignored by the researchers. The lack of information on the correlation between the plant disease incidence and pedoclimatic factors hindered our ability to develop more sustainable management strategies for many plant diseases [103]. The latter continue to cause large losses on a wide range of important crops because management practices, including the use of genetic resistance, are not complete and in many cases, sole reliance on chemical products has led to pathogen resistance in the field for many species in the genus [104]–[110]. This study demonstrates how the knowledge of pedoclimatic factors could clearly lead to better understand the role of different factors on plant disease occurrence and spread.

Geostatistic analysis showed significant correlation among the DI and investigated pedoclimatic parameters, from geostatistical point of view. Disease incidence appeared correlated to higher values of annual rainfall, a higher content of nitrogen in the soil, higher thermal shock values and a lower Mg/K ratio in the soil. Additionally, a weak positive and negative correlations of soil aluminium and plant age, respectively, were detected with DI. However, The last two factors have less impact on disease occurrence and development compared to other pedoclimatic variables investigated. The significance of these correlations is likely the consequence on the infection process and spread of the disease.

Higher nitrogen level in contributing disease development [77], [111] is of vital concern. However, it must be emphasized that the higher nitrogen levels found across our study sites, are not high, in absolute terms and might not be due to the natural presence of this trophic element. Especially, large amounts of mineral nitrogen, indiscriminately used in the last five decades, in the name of intensive agriculture might have unbalanced significantly the nitrogen content in the soil. The return to balanced fertilization practices with regard to nitrogen may well be in favor of a restoration of balance that was lacking for long time. Previously, a possible problem caused by the unbalanced fertilizations, as a cause of increased occurrence and spread of hazelnut diseases in the studied area, was hypothesized [112].

High coefficient of determination value confirmed a strong correlation among the average rainfall and DI. Rainfall is considered one of the most influencing factors on phytobacterial disease occurrence [113]. Since Xac has the epiphytic phase on hazelnut plants [9], [10], probably, it becomes airborne in splash droplets during the rainfall, as other epiphytic bacteria [114]. In addition, the importance of rainfall in triggering the multiplication of the epiphytes and the consequence for the epidemiology of bacterial diseases of plants have been demonstrated [115]. Once bacteria multiply on the phylloplane high levels of bacterial populations can be reached. As a consequence, during the rainfall period (common across our study sites), the bacterial pathogen spreads at a rate that might cause severe infection, as bacteria within lesions are released very readily from wet leaves [116]–[119]. Moreover, the potential number of cells available for dispersal from a single spot on a single infected leaf may be greater than the entire epiphytic population on the surfaces of several thousand leaves or plants [78]. Previous reports suggested that the presence of leaf symptoms, caused by Xac, are rare in orchards [9], [10]. In contrast to that report, we found very frequent presence of leaf symptoms across the study sites, especially in certain areas where the DI was almost 75%. Since hazelnut cultivation is practiced within a very concentrated area of Viterbo Province, the pathogen can easily disperse through the rainfall rapidly across the entire cultivation sites. These considerations might turn very useful in order to take preventive measures in controlling disease occurrence.

In addition to the key role, rainfall might play a secondary role in causing disease occurrence across our study sites. The presence of compact soil is common in the area due to the lack of soil breaking up practices [120]. As the consequence, soil aeration, drainage and the consequent root elongation did not occur. Root asphyxia phenomenon is very frequent during the major raining seasons, causing plants general suffering. All these stress conditions predispose plants to bacterial attacks [121].

The relationship between the DI and lower values of Mg/K ratio is more difficult to interpret. No study in literature is available in this regard. However, this might be partly explained by the fact that magnesium is an antagonist of potassium or *vice versa* because of the competition between the ions [122]–[125]. Since Mg^2+^ and K^+^ as Ca^2+^ are similar in size and charge, exchange sites cannot distinguish the difference between them. An indiscriminate acceptance of either of these ions (the most abundant), at the expense of other, at the exchange sites, is the consequence. Moreover, the binding strengths of K^+^ is much stronger than Mg^2+^ and for this the first easily out-competes the second at exchange sites [122]. The presence at very high concentration of one of these ions results in a complete suppression in uptake mechanism of the other [124]–[131]. It is well established that most of our study sites has the soils of volcanic origin, rich of potassium [132], confirming the reason of lower Mg/K ratio. Magnesium is one of the essential elements for plant growth and production [133], [134] and its deficiency renders plants susceptible predisposing them to the disease.

A strong correlation is likely to show the role of thermal shock on the DI. The higher thermal shock values, especially those referred to the critical periods (presence of night frost), damage the plant tissues and create the micro-lesions. The latter represent an ideal route of entry for bacteria and the consequent infection begin process. Recent studies showed higher thermal shock values registered across Viterbo province [84], [96] with consequent effects on the health status of hazelnut plants.

In addition to the parameters described above, the role of soil aluminium (Al) content associated to lower pH values might play an important role in disease occurrence. Previous studies showed the presence of acidic soil (pH<5) across some of our study sites [120], [135] which are much lower than the optimal pH values (5.5–7.8), for hazelnut crop [136]. Soils with lower pH values can increase the susceptibility of fruit tree species to bacterial diseases, especially to those caused by pseudomonads [81]–[83]. In addition, lower pH values result fatal in association to higher Al in soil. This metal can be present in different forms in the soil. The trivalent A1 form, (A1^3+^), dominates in acid condition (pH<5) which is toxic to plants [137]. Poor root elongation, growth reduction and premature aging of the plants are the consequence [138], [139]. The phytopathogenic bacteria, in these circumstances, easily explicate their virulence causing the disease. However, we did not find any significant correlation among the low pH, high aluminium soil values and the incidence of bacterial blight. Pearson correlation coefficient matrix, among soil pH and aluminium, further confirmed no significant correlation with the DI. This is probably because acidic soils do not increase host susceptibility to diseases caused by Xanthomonads, unlike those described for pseudomonads. Nonetheless, the poor correlation of the DI to soil aluminium values is also affected by the relatively high CVPE values of the model applied.

Factors that depend strictly by agronomic practices (total nitrogen and Mg/K ratio of soil), can be improved on behalf of crop requirement. Nevertheless the negative effect of rainfall and thermal shock are not easily manageable. In this context, the potential effects (direct and indirect) of climate change should not be overlooked. Increased atmospheric CO_2_, heavy and unseasonal rains, increased humidity, drought, cyclones and hurricanes and warmer winter temperature are the major climate change factors influencing disease occurrence, severity and spread [25], [140]–[144]. Changes to any one of these climatic factors can affect the distribution and biology of plant pathogens with very serious economic consequences [145], [146]. Several studies hypothesized the increasing CO_2_ as the main cause of changes in crop architecture, leading to increased humidity within the canopy and more suitable condition for pathogen survival [28], [147]. In addition, increased photosynthetic rate, under elevated CO_2_ levels [146] might lead to the availability of new growth flushes earlier in the season for pathogen to colonize and multiply in [27]. Changes in rainfall pattern and temperature might affect the epidemiology of plant diseases including the survival of primary inoculums [148], the rate of disease progress and even the duration of epidemics [27]. These phenomena might be favorable for growth, multiplication and spread also of hazelnut bacterial blight pathogen.

The results presented here confirm the role of some pedoclimatic factors in the occurrence and spread of hazelnut bacterial blight disease. This is the first epidemiological study, based on detailed data, on this disease from central Italy. Improved crop management, through adequate agronomic techniques, is possible across our study sites, especially in the areas where the risk of bacterial blight is higher. Detailed information on biology, epidemiology and control of Xac are described [7], [8], [9], [10]. This information is essential for the effective control of the pathogen. However, besides the general control measures, the disease strictly related to pedoclimatic conditions, at local level requires specific control measures. Referring to our study sites, improvement of soil drainage system, through soil breaking up practices might be necessary. The latter might be sufficient once every five years, provided that it is done at greater depth, without compromising mechanical harvest. The application of copper-based compounds, the only chemicals allowed in Italy, should be done following the phenomena that cause lesions on plants (pruning, spring frost and hail). Balanced nitrogen fertilization is essential across our study sites. Increased use of mineral fertilizers, in the last five decades, caused serious problems in soil mineral equilibrium. Lower soil pH values in the area are attributed to the indiscriminate use of soil acidifying nitrogen fertilizers [135]. Reduction in use of mineral nitrogen and increased use of organic substances must be done. Soil liming is advisable in areas where pH values are particularly lower to avoid plant stress [136]. An increase in pH values in these soils can result in increased absorption of calcium, magnesium and potassium [138]. Finally, the problem of lower Mg/K ratio can be addressed by the application of foliar fertilization of magnesium salts.

## Supporting Information

Appendix S1 Statistical tests, Pearson correlation table and multiple regression analysis of the disease incidence with pedoclimatic factors and plant age.
(DOCX)

Appendix S2 Transformation of pedoclimatic variables.
(DOCX)

Appendix S3 Maps and correlation graphs of disease incidence with soil aluminium, pH and plant age.
(DOCX)

Figure S1 Reaction observed on the bean pods inoculated with bacteria. Pods inoculated with strains of *Pantoea agglomerans* (A) and *Xanthomonas arboricola* pv. *corylina* (B). In the first case, the tissues of the pods inoculated with *P. agglomerans* did not collapse (C) whereas tissue collapsing was observed in the second case (D). Figures A and B are referred to the naked-eye observation whereas stereomicroscope (Stemi DV4) observation was made for C and D (5 X).
(TIF)

Figure S2 Characteristic symptoms of bacterial blight developed on the artificially inoculated hazelnut plant. Water-soaked necrotic spots on the leaves (A), shoot dieback (B) and canker formation (C) observed respectively at 3, 4 and 5 weeks after inoculation. Figures are referred to 2-year old potted plants (cv. Tonda Gentile Romana).
(TIF)

Table S1 Study areas, hazelnut cultivars, plant age and bacterial blight incidence across the Viterbo province.
(DOCX)

## References

[1] FAO statistics (2010) Available: http://www.faostat.fao.org/site/339/default.aspx. Accessed 2012 Feb 9.

[2] Me G, Valentini N (2006) La corilicoltura in Italia e nel mondo. Petria 16: 7–18.

[3] Pedica A, Vittori D, Ciofo A, De Pace C, Bizzarri S, et al (1997) Evaluation and utilization of *C. avellana*. Genetic resources to select clones for hazelnut varietal turnover in the Latium region (Italy). Acta Hort 445: 123–134.

[4] Vauterin L, Hoste B, Kersters K, Swings J (1995) Reclassification of *Xanthomonas* . Int J Syst Bacteriol 45: 472–489.

[5] Barss HP (1913) A new filbert disease in Oregon. Oregon Agricultural Experiment Station Biennial Crop Pest and Horticulture. Rep 14: 213–223.

[6] Bradbury JF (1987) *Xanthomonas campestris* pv. *corylina*. CMI Descriptions of Pathogenic Fungi and Bacteria No. 896. CAB International, Wallingford, UK.

[7] OEPP/EPPO (1986) Data sheet on quarantine organisms, 134: *Xanthomonas campestris* pv. *corylina* (Miller et al. 1940) Dye 1978. OEPP/EPPO Bull 16: 13–16.

[8] OEPP/EPPO (1990) Specific quarantine requirements. EPPO Technical Documents No. 1008.

[9] OEPP/EPPO (2004) Diagnosis protocols for regulated pests *Xanthomonas arboricola* pv. *corylina* . OEPP/EPPO Bull 179: 179–181.

[10] OEPP/EPPO (2004) Diagnosis protocols for regulated pests *Xanthomonas arboricola* pv. *corylina* . OEPP/EPPO Bull 34: 155–157.

[11] Kazempour MN, Ali B, Elahinia SA (2006) First report of bacterial blight of hazelnut caused by *Xanthomonas arboricola* pv. *corylina* in Iran. J Plant Pathol 88: 341.

[12] Poschenrieder G, Czech I, Friedrich-Zorn M, Huber B, Theil S, et al. (2006) Ester nachweiss von *Pseudomonas syringae* pv. *coryli* (pv. nov.) und *Xanthomonas arboricola* pv. *corylina* an *Corylus avellana* (Haselnuss) in Deutschland. Bayerische Landesanstalt fur Landwirtschaft-Insitut fur Pflanzenschutz. Jahrb pp. 32–33.

[13] Pulawska J, Kaluzna M, Kolodziejska A, Sobiczewski P (2010) Identification and characterization of *Xanthomonas arboricola* pv. *corylina* causing bacterial blight of hazelnut: a new disease in Poland. J Plant Pathol 92: 803–806.

[14] Lamichhane JR, Grau P, Varvaro L (2012) Emerging hazelnut cultivation and the severe threat of bacterial blight in Chile. J Phytopathol doi:10.1111/jph.12004.

[15] Petri L (1932) Rassegna dei casi fitopatologici osservati nel 1931. Bollettino R. stazione Pat. Veg Roma 12: 1–64.

[16] Petri L (1933) Rassegna dei casi fitopatologici osservati nel 1931. Bollettino R. stazione Pat. Veg Roma 13: 1–73.

[17] Noviello C (1968) Osservazioni sulle malattie parassitarie del nocciolo con particolare riferimento alla Campania. Annali della Facoltà di Scienze Agrarie dell'Università di Napoli, Portici. Ann 4: 3–31.

[18] Scortichini M, Rossi MP (1991) Presenza endemica di *Xanthomonas campestris* pv. *corylina* in noccioleti del Lazio. Inf Fitopatol 41: 251–256.

[19] Fiori M, Loru L, Marras PM, Virdis S (2006) Le principali avversità del nocciolo in Sardegna. Petria 16: 71–88.

[20] Siscaro G, Longo S, Catara V, Cirvilleri G (2006) Le principali avversità del nocciolo in Campania. Petria 16: 59–70.

[21] Virdis S (2008) Studio delle principali malattie del nocciolo in Sardegna. Phd Thesis, University of Sassari, Sardegna, Italy. 72 p.

[22] Varvaro L (1993) Le fitopatie del nocciolo nell'alto Lazio: un triennio di osservazioni e di strategie di lotta. Inf Fitopatol 2: 54–58.

[23] Lamichhane JR, Fabi A, Varvaro L (2012) Severe outbreak of bacterial blight caused by *Xanthomonas arboricola* pv. *corylina* on hazelnut, cv. Tonda di Giffoni, in central Italy. Plant Dis 96: 1577. 10.1094/PDIS-04-12-0375-PDN 30727324

[24] Mazzone P, Ragozzino A (2006) Le principali avversità del nocciolo in Campania. Petria 16: 19–30.

[25] Chakraborty S, Newton AC (2011) Climate change, plant diseases and food security: an overview. Plant Pathol 60: 2–14.

[26] Fitt BDL, Fraaije BA, Chandramohan P, Shaw MW (2011) Impacts of changing air composition on severity of arable crop disease epidemics. Plant Pathol 60: 44–53.

[27] Luck J, Spackman M, Freeman A, Trebicki P, Griffiths W, et al (2011) Climate change and diseases of food crops. Plant Pathol 60: 113–121.

[28] Pangga R, Hanan J, Chakraborty S (2011) Pathogen dynamics in a crop canopy and their evolution under changing climate. Plant Pathol 60: 70–81.

[29] Broders KD, Wallhead MW, Austin GD, Lipps PE, Paul PA, et al (2009) Association of soil chemical and physical properties with *Pythium* species diversity, community composition, and disease incidence. Phytopathology 99: 957–967. 19594315 10.1094/PHYTO-99-8-0957

[30] Duffy BK, Ownley BH, Weller DM (1997) Soil Chemical and Physical Properties Associated with Suppression of Take-all of Wheat by *Trichoderma koningii* . Phytopathology 87: 1118–1124. 18945008 10.1094/PHYTO.1997.87.11.1118

[31] Miller PW (1949) Filbert bacteriosis and its control. Oregon Agricultural Experiment Station Technical Bulletin No. 6.

[32] Campbell CL, Noe JP (1985) The spatial analysis of soil-borne pathogens and root diseases. Annu Rev Phytopathol 23: 129–148.

[33] Chellemi DO, Rohrbach KJ, Yost RS, Sonoda RM (1988) Analysis of the spatial pattern of plant pathogens and diseased plants using geostatistics. Phytopathology 78: 221–226.

[34] Gent DH, Farnsworth JL, Johnson DA (2011) Spatial analysis and incidence-density relationships for downy mildew on hop. Plant Pathol 61: 37–47.

[35] Henne DC, Workneh F, Rush CM (2012) Spatial patterns and spread of potato zebra chip disease in the Texas Panhandle. Plant Dis 96: 948–956. 10.1094/PDIS-09-11-0805-RE 30727220

[36] Orum TV, Bigelow DM, Nelson MR, Howell DR, Cotty PJ (1997) Spatial and temporal patterns of *Aspergillus flavus* strain composition and propagule density in Yuma County, Arizona, soils. Plant Dis 81: 911–916. 10.1094/PDIS.1997.81.8.911 30866380

[37] Ferrin DM, Mitchell DJ (1986) Influence of initial density and distribution of inoculums on the epidemiology of tobacco black shank. Phytopathology 76: 1153–1158.

[38] Gray SM, Moyer JW, Bloomfield P (1986) Two dimensional distance class model for quantitative description of virus-infected plant distribution lattices. Phytopathology 76: 243–248.

[39] Madden LV, Louie R, Abt JJ, Knoke JK (1982) Evaluation of tests for randomness of infected plants. Phytopathology 72: 195–198.

[40] Martins L, Castro J, Macedo W, Marques C, Abreu C (2007) Assessment of the spread of chestnut ink disease using remote sensing and geostatistical methods. Eur J Plant Pathol 119: 159–164.

[41] Nicot PC, Rouse DI, Yandell BS (1984) Comparision of statistical methods for studying spatial patterns of soilborne plant pathogens in the field. Phytopathology 74: 1399–1402.

[42] Noe JP, Campbell CL (1985) Spatial pattern analysis of plant parasite nematodes. J Nematol 17: 86–93. 19294064 PMC2618431

[43] Proctor CH (1984) On the detection of clustering and anisotrophy using binary data from a lattice patch. Commun Stat Theror Meth 13: 617–638.

[44] Ramirez BN, Michell DJ (1975) Relationship of density of chlamydospores and zoospores of *Phytopthora palmivora* in soil to infection of papaya. Phytopathology 65: 780–785.

[45] Clark I (1979) Pratical Geostatistics. Elsever Applied Science Publishers, Essex, England. 129 p.

[46] Cliff AD, Ord JK (1977) Spatial Autocorrelation. Pion, London, 178 p.

[47] Goodchild MF (1993) The state of GIS for environmental problem solving. In: Goodchild M F, Parks BO, Steyaert LT, editors. Environmental Modeling with GIS. Oxford University Press, London. pp. 8–15.

[48] Nelson MR, Felix-Gastelum R, Orum TV, Stowell LJ, Myers DE (1994) Geographic information systems and geostatistics in the design and validation of regional plant virus management programs. Phytopathology 84: 898–905.

[49] Nelson MR, Orum TV (1997) Geographic information systems and geostatistics in the design of regional plant disease and insect pest management programs. AAAS Annu Meet Sci Innov Expo 163: A22.

[50] Nelson MR, Orum TV, Jaime-Garcia R, Nadeem A (1999) Applications of geographic information systems and geostatistics in plant disease epidemiology and management. Plant Dis 83: 308–319. 10.1094/PDIS.1999.83.4.308 30845581

[51] Orum TV, Bigelow DM, Nelson MR, Howell DR, Cotty PJ (1997) Spatial and temporal patterns of *Aspergillus flavus* strain composition and propagule density in Yuma County, Arizona, soils. Plant Dis 81: 911–916. 10.1094/PDIS.1997.81.8.911 30866380

[52] Wu BM, Van Bruggen AHC, Subbarao KV, Pennings GGH (2001) Spatial analysis of lettuce downy mildew using geostatistics and geographic information systems. Phytopathology 91: 134–142. 18944386 10.1094/PHYTO.2001.91.2.134

[53] Broders KD, Wallhead MW, Austin GD, Lipps PE, Paul PA, et al (2009) Association of soil chemical and physical properties with *Pythium* species diversity, community composition, and disease incidence. Phytopathology 99: 957–967. 19594315 10.1094/PHYTO-99-8-0957

[54] Martins LM, Oliveira MT, Abreu CG (1999) Soils and climatic characteristic of chestnut stands that differ on the presence of the Ink Disease. Acta Hort 494: 447–449.

[55] Martins LM, Lufinha MI, Marques CP, Abreu CG (2001) Small format aerial photography to assess Chestnut Ink Disease. For Snow Landsc Res 73: 357–360.

[56] Martins LM, Macedo FW, Marques CP, Abreu CG (2005) Assessment of Chestnut Ink Disease spread by geostatistical methods. Acta Hort 693: 621–625.

[57] Gottwald TR, Avinent L, Llácer G, de Mendoza Hermoso A, Cambra M (1995) Analysis of the spatial spread of sharka (Plumb pox virus) in apricot and peach orchards in eastern Spain. Plant Dis 79: 266–278.

[58] Larkin RP, Gumpertz ML, Ristaino JB (1995) Geostatistical analysis of *Phytophthora* epidemic development in commercial bell pepper fields. Phytopathology 85: 191–203.

[59] Stein A, Kocks CG, Zadoks JC, Frinking HD, Ruissen MA, et al (1994) A geostatistical analysis of the spatio-temporal development of the downy mildew epidemics in cabbage. Phytopathology 84: 1227–1239.

[60] Eastman JR (2001) Guide to GIS and Image Processing. Vol I, Clark University Worcester, MA USA. 171 p.

[61] Eastman JR (2001) Guide to GIS and Image Processing. Vol II, Idrisi Release Worcester, MA:Clark University. 144 p.

[62] Deutsch CV, Journel AG (1992) GSLIB, Geostatistical Software Library and Users Guide. New York: Oxford Press. 340 p.

[63] Yamamoto JK (1999) Quantification of Uncertainty in Ore-Reserve Estimation: Applications to Chapada Copper Deposit, State of Goias, Brazil. Natural Res J 8: 153–163.

[64] Star J, Estes JE (1990) Geographic Information Systems: An Introduction. Prentice Hall, Englewood.Cliffs, New Jersey. 303 p.

[65] Gardan L (1986) *Xanthomonas campestris* pv. *corylina*. EPPO Data sheets on quarantine organisms. EPPO/EPO Bull 16: 13–16.

[66] Gardan L, Devaux N (1983) Bacterial blight of hazelnut caused by *Xanthomonas corylina* . Proc. International congress on hazelnut, Avellino, Italy 443–450.

[67] Seem RC (1984) Disease incidence and severity relationships. Ann Rev Phytopathol 22: 133–150.

[68] Lee YA, Hildebrand DC, Schroth MN (1992) Use of quinate metabolism as a phenotypic property identify members of *Xanthomonas campestris* dna homology group 6. Phytopathology 82: 971–973.

[69] Lamichhane JR, Fabi A, Varvaro L (2012) Bacterial species associated to brown spots of hazelnut in central Italy: Survey, isolation and characterization. Acta Hort In press.

[70] Lelliot RA, Stead DE (1987) Methods for the diagnosis of bacterial diseases of plants. In: Preece TF editor. Methods in Plant Pathology, Volume 2. Blackwell Scientific Press, London, UK. 216 p.

[71] Schaad NW, Jones JB, Chun W (2001) Laboratory Guide for the Identification of Plant Pathogenic Bacteria. Third edition, 373 p.

[72] Ayers SH, Rupp P, Johnson WT (1919) A study of the alkali forming bacteria in milk. USDA Bull 882.

[73] Klement Z, Lovrekovich L (1961) Defence Reactions Induced by Phytopathogenic Bacteria in Bean Pods. J Phytopathol 41: 217–227.

[74] Klement Z, Goodman RN (1967) The hypersensitive reaction to infection by bacterial plant pathogens. Ann Rev Phytopathol 5: 17–44.

[75] Roberts SJ (1997) Effect of weather conditions on local spread and infection by pea bacterial blight (*Pseudomonas syringae* pv. *pisi*). Eur J Plant Pathol 103: 711–719.

[76] Agrios GN (2005) Plant Pathology. Amsterdam: Elsevier-Academic Press. 948 p.

[77] Balestra GM, Varvaro L (1997) Influence of nitrogen fertilization on the colonization of olive phylloplane by *Pseudomonas syringae* subsp. *savastanoi* In: Rudolph K, Burr TJ, Mansfield JW, Stead D, Vivian A, et al. editors. *Pseudomonas syringae* Pathovars and Related Pathogens. Kluwer Academic Publishers, Dordrecht, The Netherlands. pp. 88–92.

[78] Shear GM, Wingard SA (1944) Some ways by which nutrition may affect severity of disease in plants. Phytopathology 34: 603–605.

[79] Barnett HL (1959) Plant disease resistance. Ann Rev Microbiol 13: 191–210.

[80] Dordas C (2008) Role of nutrients in controlling plant diseases in sustainable agriculture. A review. Agron Sustain Dev 28: 33–46.

[81] Melakeberhan H, Jones AL, Hanson E, Bird GW (1995) Effect of low soil pH on aluminium availability and on mortality of cherry seedlings. Plant Dis 79: 886–892.

[82] Vigoroux A, Bussi C (1993) Influence of water availability and soil calcic amendment on susceptibility of apricot to bacterial canker. Acta Hort 384: 607–611.

[83] Weaver DJ, Wehunt EJ (1975) Effect of soil pH on susceptibility of peach to *Pseudomonas syringae* . Phytopathology 65: 984–989.

[84] Fabi A, Varvaro L (2009) Application of geostatistics in studying epidemiology of hazelnut diseases: a case study. Acta Hort 845: 507–514.

[85] Fabi A, Varvaro L (2006) Spatial and temporal distribution of dieback of hazelnut on Cimini hills (Central Italy) by use of Geographic Information System and Geostatistics. Proc. 12th Congr. Medit. Phytopath. Union, June 11–15, Rhodes Island (Greece), 217–219.

[86] Tangmar BB, Yost RS, Uehara G (1985) Application of geostatistics to spatial studies of soil properties. Adv Agron 38: 45–94.

[87] Guo XD, Fu BJ, Ma KM, Chen LD (2000) Spatial variability of soil nutrients based on geostatistics combined with GIS – A case study in Zunhua City of Hebei Province. Study on spatial variation of Chinese J Appl Ecol 11: 557–563 (in Chinese). 11767677

[88] Cambardella CA, Moorman TB, Parkin TB, Karlen DL, Novak JM, et al (1994) Field-scale variability of soil properties in central Iowa soils. Soil Sci Soc Am J 58: 1501–1511.

[89] Oliver MA, Webster R (1990) Kriging: a method of interpolation for geographical information systems. Int J Geogr Inf Syst 4: 313–332.

[90] Cressie N (1985) Fitting variogram models by weighted least squares. Math Geol 17: 563–586.

[91] Cressie N (1988) Spatial Prediction and Ordinary Kriging. Math Geol 20: 405–421. Erratum, (1989). Math Geol 21: 493–49.

[92] Boccacci P, Torello MD, Botta R, Rovira M (2009) Genetic diversity and relationships among Italian and Spanish hazelnut cultivars. Acta Hort 845: 127–132.

[93] Tombesi A, Limongelli F (2002) Varietà e miglioramento genetico del nocciolo. Atti del Convegno Nazionale sul Nocciolo, le frontiere delle corilicoltura italiana. Giffoni Valle Piana (SA), 5–6 ottobre; pp. 11–27.

[94] Cristofori V, Bignami C, De Salvador R, Rugini E (2011) Il nocciolo in Italia: valorizzazione del prodotto e innovazione colturale per garantire competitività. Frutticol 5: 44–53.

[95] Scortichini M, Rossi MP, Marchesi U (2002) Genetic, phenotypic and pathogenic diversity of *Xanthomonas arboricola* pv. *corylina* strains question the representative nature of the type strain. Plant Pathol 51: 374–381.

[96] Fabi A, Belli C, Vuono G, Balestra GM, Varvaro L (2005) Innovative strategies in epidemiological studies of Hazelnut dieback by using G.P.S./G.I.S. and A.Sp.I.S. Technology. Acta Hort 686: 427–433.

[97] Isaaks EH, Srivastava RM (1989) An Introduction to Applied Geostatistics. Oxford University Press, New York. 561 p.

[98] Goovaerts P (1997) Geostatistics for Natural Resources Evaluation. Oxford University Press, New York. 483 p.

[99] Stein ML (1999) Interpolation of Spatial Data. Some Theory for Kriging. Springer, New York. 247 p.

[100] Franke J, Gebhardt S, Menz G, Helfrich HP (2009) Geostatistical analysis of the spatiotemporal dynamics of powdery mildew and leaf rust in wheat. Phytopathology 99: 974–984. 19594317 10.1094/PHYTO-99-8-0974

[101] Jaime-Garcia R, Orum TV, Felix-Gastelum R, Trinidad-Correa R, VanEtten HD, et al (2001) Spatial analysis of *Phytophthora infestans* genotypes and late blight severity on tomato and potato in the Del Fuerte Valley using geostatistics and geographic information systems. Phytopathology 91: 1156–1165. 18943330 10.1094/PHYTO.2001.91.12.1156

[102] Van Maanen A, Xu XM (2003) Modelling plant disease epidemics. Eur J Plant Pathol 109: 669–682.

[103] Ristiano JB, Gumpertz ML (2000) New frontiers in the study of dispersal and spatial analysis of epidemics caused by species in the genus phytopthora. Annu Rev Phytopathol 38: 541–576. 11701854 10.1146/annurev.phyto.38.1.541

[104] Brent KJ, Hollomon DW (1998) Fungicide Resistance: The Assessment of Risk.Brussels, Belgium: Global Crop Prot Fed 48 p.

[105] Cooksey DA (1990) Genetics of bactericide resistance in plant pathogenic bacteria. Ann Rev Phytopathol 28: 201–219.

[106] Genet JL, Jaworska G, Deparis F (2006) Effect of dose rate and mixtures of fungicides on selection for QoI resistance in populations of *Plasmopara viticola* . Pest Manag Sci 62: 188–194. 16411165 10.1002/ps.1146

[107] Gisi U, Waldner M, Kraus N, Dubuis PH, Sierotzki H (2007) Inheritance of resistance to carboxylic acid amide (CAA) fungicides in *Plasmopara viticola* . Plant Pathol 56: 199–208.

[108] Martin HL, Hamilton VA, Kopittke RA (2004) Copper tolerance in Australian populations of *Xanthomonas campestris* pv. *vesicatoria* contributes to poor field control of bacterial spot of pepper. Plant Dis 88: 921–924. 10.1094/PDIS.2004.88.9.921 30812242

[109] Sudin GW, Bender CL (1993) Ecological and genetic analysis of copper and streptomycin resistance in *Pseudomonas syringae* pv. *syringae* . Appl Environ Microbiol 59: 1018–1024. 8476279 10.1128/aem.59.4.1018-1024.1993 PMC202231

[110] Vanneste JL, McLaren GF, Yu J, Cornish DA, Boyd R (2005) Copper and streptomycin resistance in bacterial strains isolated from stone fruit orchards in New Zealand. N Z Plant Prot 58: 101–105.

[111] Snoeijer SS, Pérez-García A, Joosten MHAJ, De Wit PJGM (2000) The effect of nitrogen on disease development and gene expression in bacterial and fungal plant pathogens. Eur J Plant Pathol 106: 493–506.

[112] Bianco M, Danise B (2002) La difesa fitosanitaria del nocciolo. Proceeding of the II National congresso on hazelnut. Giffoni, Valle Piana, Italy. pp. 52–61.

[113] Pietrarelli L, Balestra GM, Varvaro L (2006) Effects of simulated rain on *Pseudomonas syringae* pv. *tomato* populations on tomato plants. J Plant Pathol 88: 245–251.

[114] Butterworth J, McCartney HA (1991) The dispersal of bacteria from leaf surfaces by water splash. J Appl Microbiol 71: 484–496.

[115] Hirano SS, Upper CD (1990) Population biology and epidemiology of *Pseudomonas syringae* . Annu Rev Phytopathology 28: 155–177.

[116] Leben C, Daft GC, Schmitthenner AF (1968) Bacterial blight of soybeans: population levels of *Pseudomonas glycinea* in relation to symptom development. Phytopathology 58: 1143–1146.

[117] Haas JH, Rotem J (1976) *Pseudomonas lachrymans* inoculum on infected cucumber leaves subjected to dew- and rain-typewetting. Phytopathology 66: 1219–1223.

[118] MilesWG, Daines RH, Rue JW (1977) Presymptomatic egress of *Xanthomonas prunii* from infected peach leaves. Phytopathology 67: 895–897.

[119] Roberts SJ (1985) Bacterial diseases of woody ornamental plants. Ph.D. thesis, The University of Leeds.

[120] Aloj B, Bartoletti F, Caporossi U, D'Errico FP, Di Dato F, et al (1987) Una moria del nocciolo di natura ignota nel Viterbese. Inf Agrario 26: 55–57.

[121] Moore LW, Lagerstedt HB, Hartmann N (1974) Stress predisposes young filbert trees to bacterial blight. Phytopathology 64: 1537–1540.

[122] Hannan JM (2011) Potassium-magnesium antagonism in high magnesium vineyard soils. Ms Theses and Dissertations. Iowa State University, USA.

[123] Jacobsen ST (1993) Interaction between Plant Nutrients: III. Antagonism between Potassium, Magnesium and Calcium. Acta Agric Scandinav 43: 1–5.

[124] Pathak AN, Kalra YP (1971) Antagonism between Potassium, Calcium and Magnesium in Several Varieties of Hybrid Corn. J Plant Nutr Soil Sci 130: 118–124.

[125] Voisin A (1963) Mineral balances of soil and mineral balances of grass. In: Thomas CC editor. Grass Tetany. 262 p.

[126] Johansson OAH, Hahlin JM (1977) Potassium/Magnesium Balance in Soil for Maximum Yield. Proc. Int. Sem. on Soil Environ. and Fert. Manage. In: Intensive Agric. Soc. Sci. Soil and Manure, pp. 487–495.

[127] Marschner H (1995) Mineral Nutrition of Higher Plants. 2nd ed., Academic Press, London. 889p.

[128] Pettiet JV (1988) The Influence of Exchangeable Magnesium on Potassium Uptake in Cotton Grown on Mississippi Delta Soils. Proceedings Beltwide Cotton Production Research Conferences. pp. 517–518.

[129] Tewari SN, Sinha MK, Mandal SC (1971) Studies on the Interrelationship among Calcium, Magnesium and Potassium in Plant Nutrition. Proc Int Symp Soil Fert Eval New Delhi 1: 317–325.

[130] Prince A, Zimmerman A, Bear FE (1947) The Magnesium-supplying Powers of 20 New Jersey Soils. Soil Sci 63: 69–78.

[131] Omar MA, El Kobbia T (1966) Some Observations on the Interrelationships of Potassium and Magnesium. Soil Sci 101: 437–440.

[132] Barbieri M, Peccerillo A, Poli G, Tolomeo L (1988) Major, trace element and Sr isotopic composition of lavas from Vico volcano central Italy and their evolution in an open system. Contrib Mineral Petrol 99: 485–497.

[133] White PJ, Brown PH (2010) Plant nutrition for sustainable development and global health. Ann Bot 105: 1073–1080. 20430785 10.1093/aob/mcq085 PMC2887071

[134] Wilkinson SR, Welch RM, Mayland HF, Grunes DL (1990) Magnesium in plants: uptake, distribution, function, and utilization by man and animals. In: Helmut S, editor. Metal Ions in Biological Systems Marcel Dekker, Inc., New York and Basel, Switzerland. pp, 33–56.

[135] Scortichini M, Sbaraglia M, Di Prospero P, Angelucci L, Petricca P, et al (2001) Moria del nocciolo nel Viterbese e terreni acidi. Inf Agrario 21: 85–88.

[136] Tombesi A (1991) Il Nocciolo. Frutticol Speciale. Reda Rome, pp. 614–630.

[137] Delhaize E, Ryan PR (1995) Aluminium Toxicity and Tolerance in Plants. Plant Physiol 107: 315–321. 12228360 10.1104/pp.107.2.315 PMC157131

[138] Foy CD, Chaney RL, White MC (1978) The Physiology of Metal Toxicity in Plants. Ann Rev Plant Physiol 29: 511–566.

[139] Kinraide TB, Ryan PR, Kochian LV (1992) Interactive effects of Al^3+^, H^+^, and other cations on root elongation considered in terms of cell-surface electrical potential. Plant Physiol 99: 1461–1468. 16669059 10.1104/pp.99.4.1461 PMC1080648

[140] Cannon R (1998) The implications of predicted climate change for insect pests in the UK, with emphasis on non indigenous species. Global Change Biology 4: 785–796.

[141] Pimentel D, McNair S, Janecka J, Wightman J, Simmonds C, et al (2001) Economic and environmental threats of alien plant, animal and microbe invasions. Agr Ecosyst Environ 84: 1–20.

[142] Rosenzweig C, Iglesias A, Yang X, Epstein P, Chivian E (2001) Climate change and extreme weather events; implications for food production, plant diseases and pests. Global change Hum Health 2: 90–104.

[143] Anderson P, Cunnigham A, Patel N, Morales F, Epstein P, et al (2004) Emerging infectious diseases of plants: pathogen pollution, climate change and agrotechnology drivers. Trends Ecol Evol 19: 535–544. 16701319 10.1016/j.tree.2004.07.021

[144] Berry P, Dawson T, Harrison P, Pearson R (2002) Modelling potential impacts of climate change on the bioclimatic envelop of species in Britain and Ireland. Global Ecol Biogeogr 11: 453–462.

[145] Coakley S, Scherm H, Chakraborthy S (1999) Climate change and plant disease management. Ann Rev Phytopathol 37: 399–426. 11701829 10.1146/annurev.phyto.37.1.399

[146] Fuhrer J (2003) Agroecosystem response to combinations of elevated CO_2_, ozone and global climate change. Agr, Ecosyst Environ 97: 1–20.

[147] Chakraborthy S, Dutta S (2003) How will plant pathogens adapt to host plant resistance at elevated CO_2_ under changing climate? New Phytol 159: 733–742. 10.1046/j.1469-8137.2003.00842.x 33873600

[148] Melloy P, Hollaway G, Luck J, Norton R, Aitken ESC (2010) Production and fitness of *Fusarium pseudograminearum* inoculums at elevated carbon dioxide in FACE. Global Change and Biology 16: 3363–3373.

