# Supplementary material for: Epidemiological Study of Hazelnut Bacterial Blight in Central Italy by Using Laboratory Analysis and Geostatistics
Source: PLoS One. 2013 Feb 12;8(2):e56298. doi: 10.1371/journal.pone.0056298 (PMC3570417; doi:10.1371/journal.pone.0056298)
Supplement: Appendix S1 — Statistical tests, Pearson correlation table and multiple regression analysis of the disease incidence with pedoclimatic factors and plant age. (DOCX) [file pone.0056298.s001.docx]

**Appendix S1.**

The results of statistical tests and p-values of the correlation of the logarithmic disease incidence (Log DI) with pedoclimatic variables and plant age are shown in Table 1 (degree of freedom = 28). The Pearson coefficients r(XY) for nitrogen, Mg/K Ratio, thermal shock, rainfall, aluminium and Plant Age are significant at a p-level of 0.001 (P<0.001). The correlation is strong positive for nitrogen, thermal shock and rainfall; strong negative for Mg/K Ratio; weak positive for aluminium and weak negative for Plant Age. Hence the null hypothesis was rejected (H0:r≠0) for all of these parameters except for the soil pH which demonstrated no correlation with DI (t=-1.10; P=0.28; r^2^=0.04).

**Table 1.** Correlation of Log DI with pedoclimatic factors and plant age

| **Variables** | **r(XY)** | **r^2^** | **t** | **p** | **Constant** | **Slope** |
| --- | --- | --- | --- | --- | --- | --- |
| **Nitrogen** | 0.956***^a^ | 0.915 | 17.34 | 0.000000 | 0.126 | 0.038 |
| **Mg/K** | -0.895*** | 0.802 | -10.64 | 0.000000 | 0.997 | -0.202 |
| **Thermal shock** | 0.911*** | 0.829 | 11.65 | 0.000000 | 12.021 | 0.515 |
| **Rainfall** | 0.909*** | 0.826 | 11.54 | 0.000000 | 1026.668 | 68.701 |
| **Aluminium** | 0.602*** | 0.363 | 3.99 | 0.000427 | 0.583 | 0.063 |
| **Soil pH** | -0.203 ^NS^ | 0.041 | -1.10 | 0.281544 | 5.522 | -0.023 |
| **Age** | -0.577*** | 0.333 | -3.74 | 0.000842 | 23.618 | -5.240 |

r(XY): Pearson correlation coefficient matrix

r^2^: coefficient of determination

t: Student’s t test value

^a^Asterisks indicate: *** correlation is significant at the 0.001 level (two tailed); ^NS^ correlation is not significant

Pearson correlation values among each pair of variables are reported in Table 2 (degree of freedom = 22). Results showed that nitrogen was negatively correlated with Mg/K ratio (P<0.001; r=-0.95) and plant age (P<0.001; r=-0.54); and positively correlated with thermal shock (P<0.001; r=-0.92), rainfall (P<0.001; r=0.95) and aluminium (P<0.001; r=-0.68). Likewise, Mg/K ratio was negatively correlated with thermal shock (P<0.001; r=-0.92), rainfall (P<0.001; r=-0.96), soil aluminium (P<0.001; r=-0.68) and positively correlated with plant age (P<0.001; r=0.62). Concerning the thermal shock, it was negatively correlated with plant age (P<0.001; r=-0.48); and positively correlated with rainfall (P<0.001; r=0.93) and soil aluminium (P<0.001; r=0.64). Similarly, the correlation of rainfall was negative with plant age (P<0.001; r=-0.59) and positive with soil aluminium (P<0.001; r=0.65). A moderate negative correlation of soil aluminium (P<0.01; r=-0.49) was observed with plant age and a weak positive correlation (P<0.05; r=0.38) was detected among soil pH and plant age. It is important to note that soil pH did not show any correlation with the pedoclimatic factors except the weak positive correlation found with plant age.

**Table 2.** Pearson correlation coefficient matrix among each pair of variables considered in this study

| **Variables** | **Log (DI)** | **Nitrogen** | **Mg/K** | **Thermal shock** | **Rainfall** | **Aluminium** | **Soil pH** | **Age** |
| --- | --- | --- | --- | --- | --- | --- | --- | --- |
| **Log (DI)** | 1 |  |  |  |  |  |  |  |
| **Nitrogen** | 0.956***^a^ | 1 |  |  |  |  |  |  |
| **Mg/K** | -0.895*** | -0.952*** | 1 |  |  |  |  |  |
| **Thermal shock** | 0.911*** | 0.955*** | -0.928*** | 1 |  |  |  |  |
| **Rainfall** | 0.909*** | 0.950*** | -0.963*** | 0.937*** | 1 |  |  |  |
| **Aluminium** | 0.602*** | 0.684*** | -0.686*** | 0.647*** | 0.657*** | 1 |  |  |
| **Soil pH** | -0.203^NS^ | -0.153 ^NS^ | 0.235 ^NS^ | -0.223 ^NS^ | -0.267 ^NS^ | -0.056 ^NS^ | 1 |  |
| **Age** | -0.577*** | -0.540*** | 0.621*** | -0.485** | -0.596*** | -0.490** | 0.388* | 1 |

r(XY): Pearson correlation coefficient matrix

^a^Asterisks indicate: * correlation is significant at the 0.05 level (two tailed) ; ** correlation is significant at the 0.01 level (two tailed); *** correlation is significant at the 0.001 level (two tailed); ^NS^ correlation is not significant

By analyzing the data in a multiple regression approach it is possible to quantify a relationship among the parameters investigated. The summary of statistics, the regression summary of the coefficients and associated significance tests for Log DI are the followings: No. of cases = 30; degree of freedom = 7.22; multiple R = 0.9675; multiple R² = 0.9361; adjusted R² = 0.9158; F (7.22) = 46.0495; P = 0.000000 (P<0.001) and Standard error of estimate = 0.2374.

**Table 3.** Multiple regression analysis of parameters investigated in this study

|  | β | βSE | **B** | **BSE** | **t(22)** | **p-level** |
| --- | --- | --- | --- | --- | --- | --- |
| **Intercept** |  |  | -4.56501 | 6.526174 | -0.69949 | 0.491575 |
| **Nitrogen** | 1.203182 | 0.249090 | 30.49941 | 6.314174 | 4.83031 | 0.000080 |
| **Mg/K ratio** | 0.369649 | 0.237591 | 1.63777 | 1.052672 | 1.55582 | 0.134020 |
| **Thermal shock** | 0.032907 | 0.205310 | 0.05822 | 0.363244 | 0.16028 | 0.874123 |
| **Rainfall** | 0.068258 | 0.233373 | 0.00090 | 0.003088 | 0.29248 | 0.772659 |
| **Aluminium** | -0.103115 | 0.076889 | -0.98983 | 0.738078 | -1.34109 | 0.193574 |
| **Soil pH** | -0.032128 | 0.064477 | -0.28670 | 0.575374 | -0.49828 | 0.623229 |
| **Plant age** | -0.138334 | 0.078976 | -0.01523 | 0.008698 | -1.75159 | 0.093775 |

β: regression coefficient; SE: standard error

The null hypothesis is: H_0_: β_i_ = 0 for all i

The alternative hypothesis is: H_1_: β_i_ ≠ 0 for at least one i

Results of multiple regression analysis are shown in Table 3. Only the regression coefficients for nitrogen were highly significant (β=1.20; t= 4.83; P<0.001). It means that the null hypothesis can be rejected for this value. By contrast, the regression coefficient for Mg/K ratio (β=0.36; t= 1.55; P=0.13), thermal shock (β=0.03; t= 0.16; P=0.87), rainfall (β=0.06; t= 0.29; P=0.77), aluminium (β=-0.10; t= -1.34; P=0.19), soil pH (β=-0.03; t= -0.49; P=0.62) and plant age (β=-0.13; t= -1.75; P=0.09) were not significant and as such the null hypothesis cannot be rejected. This means that there is no convincing evidence that all the parameters, except average soil nitrogen, add to the predictability of DI once the other parameters are known.
