# Supplementary material for: Epidemiological Study of Hazelnut Bacterial Blight in Central Italy by Using Laboratory Analysis and Geostatistics
Source: PLoS One. 2013 Feb 12;8(2):e56298. doi: 10.1371/journal.pone.0056298 (PMC3570417; doi:10.1371/journal.pone.0056298)
Supplement: Appendix S2 — Transformation of pedoclimatic variables. (DOCX) [file pone.0056298.s002.docx]

**Appendix S2: Transformation of pedoclimatic variables**

Statistical characteristics of some pedological data showed a certain leptokurtic distribution (Table 1). The kurtosis and skewness values can be reduced by applying log-transformation (Log). However, the distribution of the climatic parameter is slightly platykurtic and not influenced by Log (data not shown). The latter can reduce the skewness, leading to a better normalized distribution of thermal shock values.

**Table 1.** Statistical characteristics of pedo-climatic parameters

| **Parameter** | **Mean** | **σ** | **Cs** | **Ck** | **Log Cs** | **Log Ck** |
| --- | --- | --- | --- | --- | --- | --- |
| **Total Nitrogen** | 0.133 | 0.055 | 1.710 | 7.260 | -0.455 | 6.935 |
| **Mg/K ratio** | 0.828 | 0.381 | 1.780 | 8.620 | -0.780 | 5.880 |
| **Δ** | 11.374 | 1.980 | 0.390 | 1.920 | 0.180 | 1.920 |
| **Aluminium** | 0.756 | 0.950 | 2.020 | 7.990 | -0.267 | 1.761 |
| **Soil pH** | 5.480 | 0.611 | 0.310 | 4.420 | -0.250 | 4.430 |
| σ: standard deviation; Cs: skewness; Ck: kurtosis; Δ: thermal shock | | | | | | |

The normal QQPlots, related to the total nitrogen in the soil, are reported as an example in figure 1. QQPlots are created by plotting data values *versus* the value of a standard normal where their cumulative distributions are equal. The difference among the QQ plot between standard normal values and the original data quantiles (Figure 1 A) and the same plot considering Log data quantiles (Figure 1 B) showed how Log data are much closer to normal distribution than the original data. The same behaviour was observed also for other pedo-cimatic parameters investigated.

**Figure 1 A:**


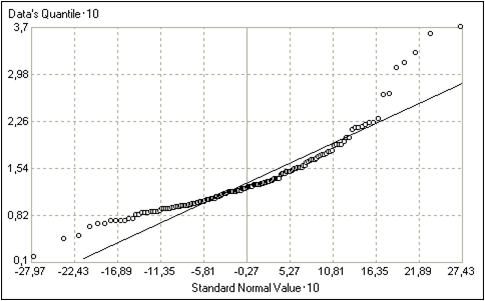


**Figure 1 B:**


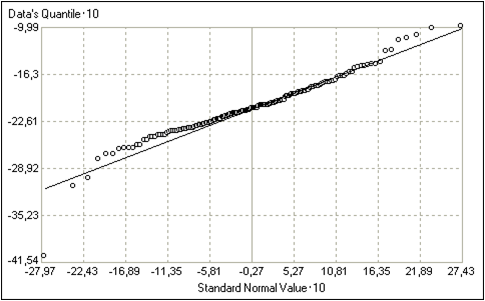


**Figure S3.** **QQ plots of total nitrogen in the soil.** Real (A) and log-transformed (B) data.
