# Supplementary material for: Epidemiological Study of Hazelnut Bacterial Blight in Central Italy by Using Laboratory Analysis and Geostatistics
Source: PLoS One. 2013 Feb 12;8(2):e56298. doi: 10.1371/journal.pone.0056298 (PMC3570417; doi:10.1371/journal.pone.0056298)
Supplement: Appendix S3 — Maps and correlation graphs of disease incidence with soil aluminium, pH and plant age. (DOCX) [file pone.0056298.s003.docx]

**Appendix S3:** Maps and correlation graphs of disease incidence with soil aluminium, pH and plant age


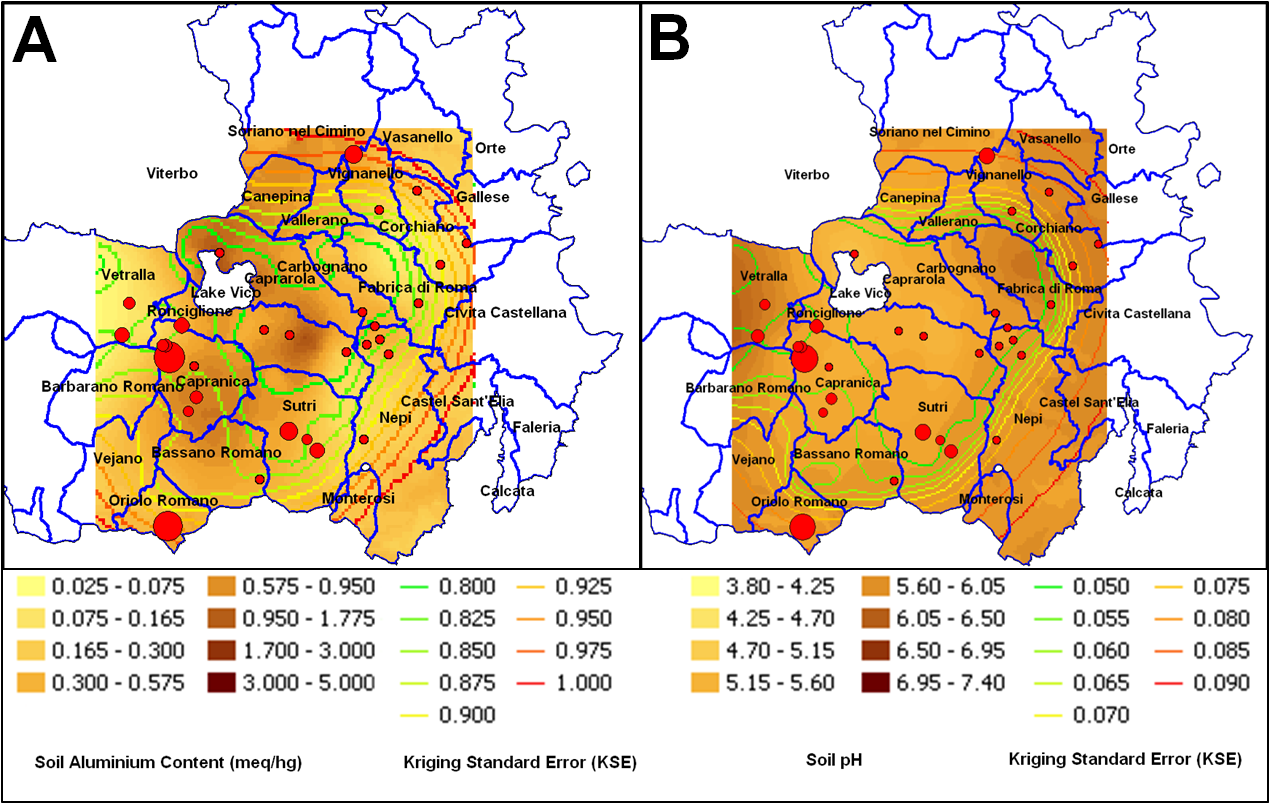


**Figure 1. Map illustrating the correlation of average disease incidence (%) with (A) soil aluminium (meq/hg) and (B) soil pH.** The red circle size inside the maps indicates the different disease incidence (<1% to 75%) expressed in logarithmic scale.

Figure 2 A:

Figure 2B:

Figure 2C:

**Figure 2. Correlation of the average disease incidence (%) of each site with different factors.** (A) average soil aluminium content (t=3.99; P<0.001; r^2^=0.36; r=0.60), (B) soil pH (t=-1.10; P=0.28; r^2^=0.04; r=-0.20) and (C) plant age (t=-3.74; P<0.001; r^2^=0.33; r=-0.57). The horizontal and vertical bars represent the standard error of dependent and independent variables, respectively. Six were the average number of replicate in each bin site (n=6).
