# Supplementary figures and images for: Epidemiological Study of Hazelnut Bacterial Blight in Central Italy by Using Laboratory Analysis and Geostatistics
Source: PLoS One. 2013 Feb 12;8(2):e56298. doi: 10.1371/journal.pone.0056298 (PMC3570417; doi:10.1371/journal.pone.0056298)

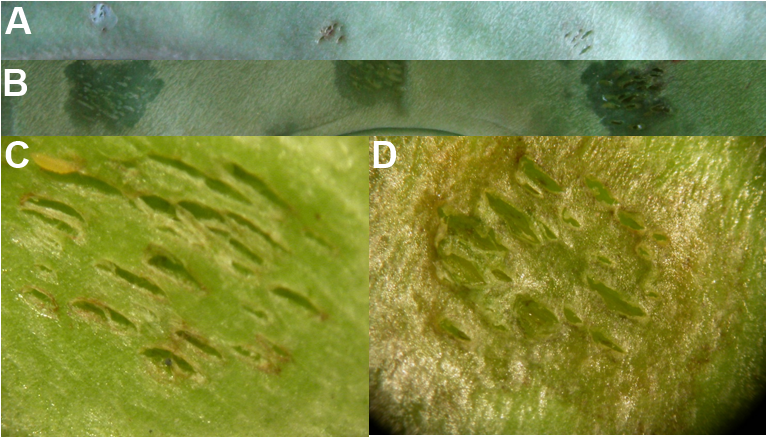

Supplement: Figure S1 — Reaction observed on the bean pods inoculated with bacteria. Pods inoculated with strains of Pantoea agglomerans (A) and Xanthomonas arboricola pv. corylina (B). In the first case, the tissues of the pods inoculated with P. agglomerans did not collapse (C) whereas tissue collapsing was observed in the second case (D). Figures A and B are referred to the naked-eye observation whereas stereomicroscope (Stemi DV4) observation was made for C and D (5 X). (TIF) [file pone.0056298.s004.tif]

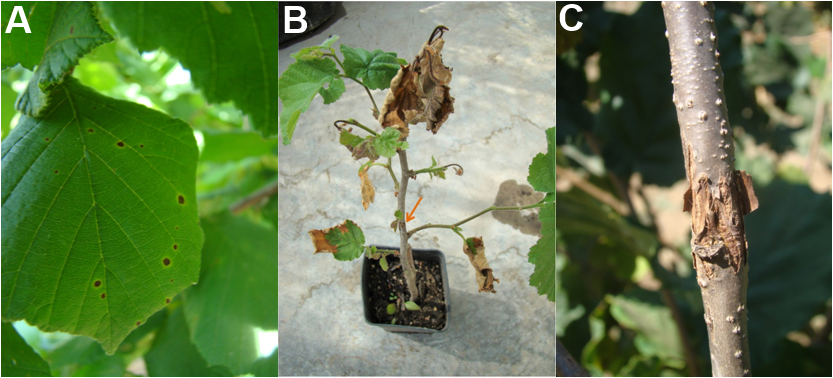

Supplement: Figure S2 — Characteristic symptoms of bacterial blight developed on the artificially inoculated hazelnut plant. Water-soaked necrotic spots on the leaves (A), shoot dieback (B) and canker formation (C) observed respectively at 3, 4 and 5 weeks after inoculation. Figures are referred to 2-year old potted plants (cv. Tonda Gentile Romana). (TIF) [file pone.0056298.s005.tif]
