# Supplementary material for: Epidemiological Study of Hazelnut Bacterial Blight in Central Italy by Using Laboratory Analysis and Geostatistics
Source: PLoS One. 2013 Feb 12;8(2):e56298. doi: 10.1371/journal.pone.0056298 (PMC3570417; doi:10.1371/journal.pone.0056298)
Supplement: Table S1 — Study areas, hazelnut cultivars, plant age and bacterial blight incidence across the Viterbo province. (DOCX) [file pone.0056298.s006.docx]

| **Municipality** | **Site** | **Cultivar^a^** | **Plant average age** | **Average incidence (%)^b^** | |
| --- | --- | --- | --- | --- | --- |
|  |  |  |  | **spots** | **canker** |
| Sutri | 1 | TGR, N | 35 | 2.17 | 0.00 |
|  | 2 | TGR, N | 30 | 6.25 | 0.00 |
|  | 3 | TGR, TDG | 25 | 12.88 | 0.00 |
| Ronciglione | 1 | TGR, N | 25 | 8.67 | 0.00 |
|  | 2 | TGR, N, TDG | 30 | 0.57 | 0.00 |
|  | 3 | TGR, N | 30 | 0.53 | 0.00 |
|  | 4 | TGR, N | 30 | 0.89 | 0.00 |
| Vetralla | 1 | TGR, TDG | 15 | 6.11 | 0.00 |
|  | 2 | TGR, N | 25 | 3.00 | 0.00 |
| Capranica | 1 | TGR, N | 30 | 2.22 | 0.00 |
|  | 2 | TGR, N | 20 | 4.00 | 0.00 |
|  | 3 | TGR, N | 4 | 74.29 | 74.29 |
|  | 4 | TGR, N | 15 | 4.00 | 0.00 |
|  | 5 | TGR, N | 15 | 3.00 | 0.00 |
|  | 6 | TGR, N | 25 | 1.00 | 0.00 |
| Oriolo Romano | 1 | TDG | 4 | 69.29 | 69.29 |
| Nepi | 1 | TGR, N | 25 | 0.65 | 0.00 |
|  | 2 | TGR, N | 30 | 0.48 | 0.00 |
|  | 3 | TGR, N | 20 | 0.44 | 0.00 |
|  | 4 | TGR, N, TDG | 20 | 0.38 | 0.00 |
|  | 5 | TGR, N | 25 | 0.28 | 0.00 |
| Vignanello | 1 | TGR, N, TDG | 25 | 0.18 | 0.00 |
| Soriano | 1 | TGR, N | 12 | 12.67 | 0.00 |
| Caprarola | 1 | TGR, N | 25 | 0.32 | 0.00 |
|  | 2 |  | 25 | 0.12 | 0.00 |
| Bassano Romano | 1 | TGR, N | 25 | 0.08 | 0.00 |
| Corchiano | 1 | TGR | 25 | 0.23 | 0.00 |
|  | 2 | TGR, TDG | 25 | 0.15 | 0.00 |
|  | 3 | TGR, TDG | 25 | 0.04 | 0.00 |
| Fabrica di Roma | 1 | TGR, N | 30 | 0.62 | 0.00 |
| Tonda Gentile Romana (TGR), Nocchione (N), Tonda Di Giffoni (TDG) | | | | | |
| ^a^Only TDG represents over 85% of the entire cultivation | | | | | |
| ^b^Values are the mean of three years and calculated by the proportion of diseased plants within the total | | | | | |
